# Critical controllability in proteome-wide protein interaction network integrating transcriptome

**DOI:** 10.1038/srep23541

**Published:** 2016-04-04

**Authors:** Masayuki Ishitsuka, Tatsuya Akutsu, Jose C. Nacher

**Affiliations:** 1 Department of Information Science, Faculty of Science, Toho University, Funabashi, 274-8510, Japan; 2 Bioinformatics Center, Institute for Chemical Research, Kyoto University, Uji 611-0011, Japan

## Abstract

Recently, the number of essential gene entries has considerably increased. However, little is known about the relationships between essential genes and their functional roles in critical network control at both the structural (protein interaction network) and dynamic (transcriptional) levels, in part because the large size of the network prevents extensive computational analysis. Here, we present an algorithm that identifies the critical control set of nodes by reducing the computational time by 180 times and by expanding the computable network size up to 25 times, from 1,000 to 25,000 nodes. The developed algorithm allows a critical controllability analysis of large integrated systems composed of a transcriptome- and proteome-wide protein interaction network for the first time. The data-driven analysis captures a direct triad association of the structural controllability of genes, lethality and dynamic synchronization of co-expression. We believe that the identified optimized critical network control subsets may be of interest as drug targets; thus, they may be useful for drug design and development.

Identifying a set of genes that are essential for sustaining life is of paramount importance for understanding the minimal genomic requirements of living cells^1^,^2^. Mutation of the essential genes, called the “*minimal gene-set*” by Koonin, can lead to a wide variety of lethal phenotypes that prevent cell survival or reproduction^3^. Because mutation of essential genes often leads to lethal phenotypes, these genes are often prioritized as drug targets and are therefore considered in drug design and development^4^. Recently, the number of essential gene entries has considerably increased. However, little is known about the relationships between essential genes and their functional roles in critical network control at both the structural (protein interaction network) and dynamic (transcriptional) levels. To address this knowledge gap, our data-driven analysis uses the latest datasets consisting of human orthologues of mouse essential genes, which are associated with 46 phenotypic categories with prenatal, perinatal and postnatal lethality^5^.

Analyses of biological networks have shown a strong correlation between the number of interactions a protein has and its lethality^6^. This protein centrality-lethality rule was recently examined from the perspective of controllability by computing the minimum dominating set (MDS) of the protein network using the method proposed in^7^ by Nacher and Akutsu. This MDS represents an identified subset of proteins, from which each remaining protein can be reached by one interaction. Therefore, members of the optimized subset of proteins act as *controllers* or *drivers*^8^ to control the remaining non-MDS proteins. The results indicated that the MDS is enriched with essential genes^9^.

Recently, research on controllability emerged as one of important topics in complex networks^8^. If a set of nodes called “driver nodes” can bring a network from any initial state to any desired state in finite time, the network is called *controllable.* Moreover if for any non-zero values of the parameters in the controllability matrices, the system is controllable, such system has structural controllability. Liu *et al.* showed in linear systems described by undirected networks that instead of computing the Kalman’s controllability condition, it is enough to compute the maximum matching in the network, and select the unmatched nodes as driver nodes. Yuan *et al.* extended these ideas by considering algebraic multiplicity and geometric multiplicity of eigenvalues that can be applied in weighted and unweighted networks, and whose predictions for driver nodes agree with those from the maximum matching algorithm^10^. On the other hand, Nacher and Akutsu showed a relationship between the Minimum Dominating Set (MDS) and structural controllability^7^, which has some resemblances with that of the edge dynamics model proposed by Nepusz and Vicsek^11^. The Nacher and Akutsu MDS-based approach was adopted by Wuchty to investigate biological aspects of the controllability in protein interaction networks^9^. If each edge in a network is bi-directional and each node in an MDS can control all of its outgoing edges individually, then the network is structurally controllable by selecting the nodes in an MDS as driver nodes. As shown in Ref. 7, there is no mathematical contradiction between Liu approach and MDS method.

In spite of the important results on protein controllability shown by Wuchty^9^, computational barriers and problems still prevented us from identifying a reliable and significant correlation between a network controller set of nodes and genomic indispensability features. First, we should note that the MDS calculation is non-trivial and represents an NP-complete decision problem in computational complexity theory^12^,^13^. Therefore, it is believed that there is no theoretically efficient algorithm that finds a dominating set of minimum cardinality for a given graph. However, Nacher and Akutsu showed that integer linear programming (ILP) can provide the exact and optimal solution for scale-free networks with more than 10,000 nodes^7^. Second, and more importantly, the solution of an MDS is not necessarily unique. There are multiple MDS configurations that can control the entire network. Each configuration denotes a different set of nodes. However, in the analysis performed by Wuchty the results consider only one of the existing multiple solutions^9^. This means that the optimized set of proteins reported in^9^ can potentially be different from that of another research group using the same dataset. Indeed, proteins can be classified into three control categories^14^,^15^. A critical set denotes those proteins that belong to every MDS configuration. A redundant set is not engaged in network control therefore, none of these proteins belongs to an MDS. An intermittent set is defined by proteins that appear in some but not in all MDSs. This feature of the MDS problem has not been investigated when associating essential roles with the optimized subset of proteins derived from the straightforward MDS computation. More importantly, according to a previous study by Nacher and Akutsu, the MDS and critical set show very different topological features, necessitating careful examination when analysing complex biological functions of proteins^15^. Recently, several computational and biological analyses were performed using MDS related techniques. However, the critical control question was left unresolved^16^,^17^,^18^,^19^,^20^,^21^. Third, the algorithm for computing the critical and redundant sets of nodes presented by Nacher and Akutsu using ILP provides an exact and optimal solution^15^. However, the algorithm needs to solve the MDS model *n* times, where *n* is the number of nodes. Therefore, compared with computing an MDS, the critical algorithmic method requires much more CPU time so the network size was limited to 1,000 nodes for most network configurations. Here, we present a new method for computing the critical set of nodes that uses the scale-freeness property of the network to elegantly circumvent the high complexity of the problem. A scale-free network is characterized by a power-law *k*^−*γ*^ for the distribution of the node degree *k.* Using a new algorithmic strategy, we could determine the critical set of proteins in protein interaction networks composed of more than 8,000 proteins (nodes) and 25,000 interactions (edges) within a few minutes, which is a remarkable computational improvement. Similarly, this algorithmic strategy was also applied to compute the redundant set of proteins in large networks. The fraction of nodes remaining after assigning critical and redundant categories can be easily classified as proteins intermittently engaged in network control. Experiments on artificially constructed scale-free networks more vividly illustrated the extent of the impact of the algorithmic improvement. As shown below, for some networks with a scale-free structure, we could analyse the MDS and the critical set with up to 25,000 nodes, and exact results could be achieved up to 180 times faster than with previously existing algorithms, an impressive improvement for obtaining exact results in an NP-hard algorithm such as an MDS-graph based problem.

The uncovered algorithmic procedure developed here allows us to analyse both the MDS and the critical control set in proteome-wide protein interaction networks for the first time. This extensive data-driven controllability analysis performed at an unprecedented scale with eight organisms provides evidence for a triad association of lethality, critical network control and biological functionality. The gene expression data also allowed us to capture a direct link between structural critical controllability of genes, gene lethality and dynamic synchronization of co-expression for the first time.

## Results

### Efficient computation of optimized critical and redundant subsets in scale-free networks

**Figure 1:**
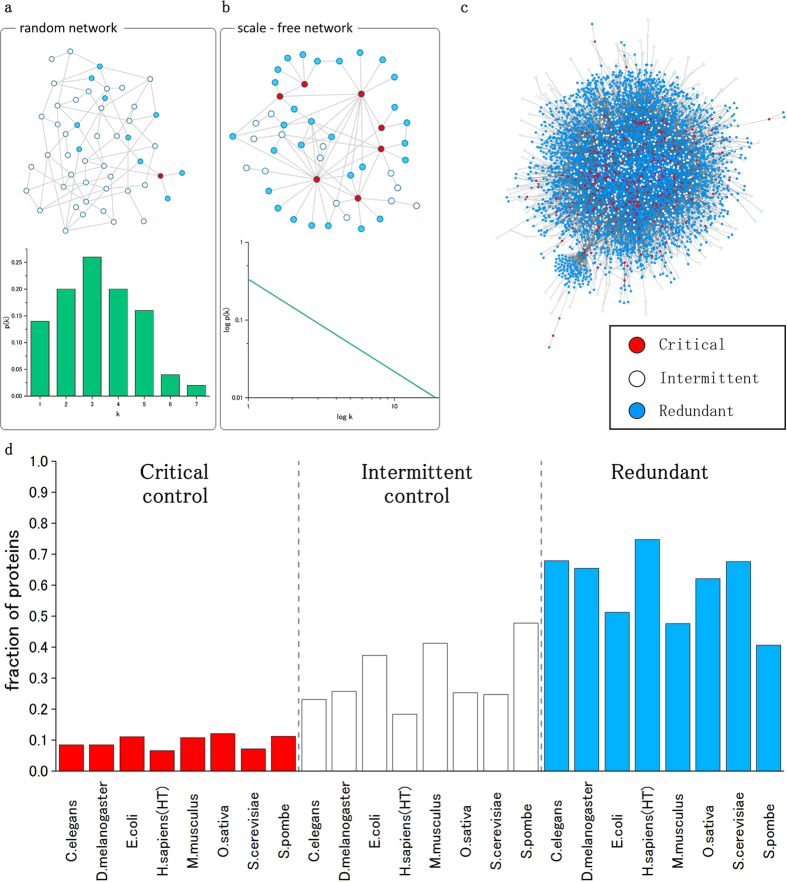
A random network (**a**) and a scale-free network (**b**) and their corresponding degree distribution. The example shows that the network structure strongly affects the controllability roles of the nodes. Whereas in random networks the critical nodes are almost absent, in scale-free networks, they have a noticeable representation impacting the controllability of the network. Random networks, by contrast, are more flexible because they depend on intermittent control nodes. The proposed algorithmic procedure relies on the structure of the scale-free networks and on the existence of highly connected nodes to pre-determine a large number of critical and redundant nodes without using ILP. (**c**) The results of the algorithm computed on the human protein interaction network. (**d**) The fraction of proteins for each control category in all analysed PPI networks. The set of proteins involved in critical control is very small (less than 10% in almost all cases). The smallest critical control set is found in the *H. sapiens* and represents 6% of all proteins.

**Figure 2:**
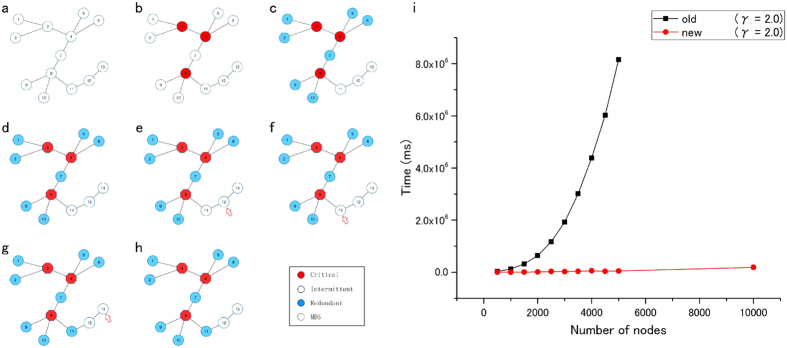
Illustration of the computation of critical, redundant and intermittent sets using the proposed algorithmic procedure. Details of the calculations, including the exact equations to be solved by ILP are shown in SI. (**a**) The initial network. Next, we apply the novel pre-processing step for determining critical nodes (**b**) and redundant nodes (**c**). (**d**) The MDS is computed for the entire network (hexagonal nodes). (**e**) Among the MDS, only node 12 is not pre-determined as a critical node. The critical set procedure is applied and node 12 (see red arrow) is determined to be intermittent. (**f–h**) The remaining nodes do not belong to the MDS; therefore, the redundant set procedure is applied sequentially to nodes 11 and 13 (see red arrow) (i) The experimental results for computational time (milliseconds (ms)) versus network size on scale-free networks with *γ* = 2 and average degree <*k*> = 2. The new algorithmic method, which pre-processes critical and redundant nodes using scale-free network features, not only clearly outperforms the computational time of previous algorithm by obtaining the exact result faster but also expands the computable network size. See also Fig. S8 in SI for the results of computational experiments results with different parameters for the constructed scale-free networks.

The novel algorithm for determining optimized sets of nodes with critical and redundant control roles has striking advantages compared with existing methods. These advances are based on two mathematical propositions that can be applied to real-world scale-free networks (see Methods for details). The network structure plays a key role in determining the control categories of the nodes. Random networks, for example, exhibit a controllability pattern that is drastically different from that of the scale-free networks (see Fig. 1(a,b)). The following two main features are essential for decreasing the complexity of the MDS and the critical set computation in scale-free networks. (1) Using algorithmic procedures developed in^15^ for determining the critical and redundant nodes in a network of size |*V|* = *n,* it is necessary to solve ILPs *n* times, in addition to the original computation for the MDS of size *|M|,* in the original network. By contrast, using the novel propositions that automatically pre-determine a subset of critical and redundant nodes, we had to solve the ILPs with a much reduced number. (2) In addition, the ILPs solved with the newly proposed method have a significantly smaller number of constraints (equations), in addition to the trivial constraints (compare Eq. 1 with Eqs 2–5 in Supplementary Information (SI)). These two features are crucial for decreasing the computational time and for identifying critical and redundant sets in large networks. Figure 2(a–f) shows an example of the new algorithm computation. For a detailed explanation, see methods section and SI. Fig. 2(i) shows the results of the computational experiments on scale-free networks with degree exponent *γ* = 2 and average degree <*k*> = 2. The result shows that the algorithm not only is able to efficiently determine the critical and redundant nodes in large networks of up to 10,000 nodes but also 180 times faster than existing algorithmic procedures. In order to examine the efficiency in more detail, we fitted the relation between the number of nodes and CPU time *t* in terms of *t* = *a* · *b*^*n*^for each of the algorithms by using Mathematica’s FindFit function after taking 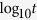 because the original problem is NP-hard and thus it is expected that CPU time grows exponentially with the number of nodes, and Fig. 2(i) also suggests such a dependency. The results were *a* = 41399.0, *b* = 1.00116 for the previous algorithm, and *a* = 4800.43, *b* = 1.00038 for the proposed algorithm, which also suggests that the proposed algorithm is much faster than the previous one. Note that difference between *b* = 1.00116 and *b* = 1.00038 is far from small because they are bases of exponential functions. Further computational experiments using scale-networks constructed with average degree <*k*> = 3 and different values for the degree exponent *γ* also confirm this improvement (see Fig. S8, in SI). In particular, in the case of scale-free networks with *γ* = 2.5 and average degree <*k*> = 3, the algorithm could compute the MDS and critical and redundant sets simultaneously for a network size of up to 25,000 nodes (see Table S3 in SI). Provided that the graph problem is NP-hard, these achievements are remarkable. The relationships with network structures from Propositions 1 and 2.2 (see Ref. 15) (see methods section) suggest that scale-free networks have the ideal structure in which the proposed algorithm can work most efficiently, as explained in the Methods section. Because most real-world networks follow a scale-free topology, in particular the analysed protein networks in this work (see Fig. S9 and Table S4), the proposed algorithm may potentially lead to multiple applications in various fields.

### The optimized subset of proteins involved in critical control is enriched with the most highly connected proteins and largely depleted of the less connected ones

**Figure 3:**
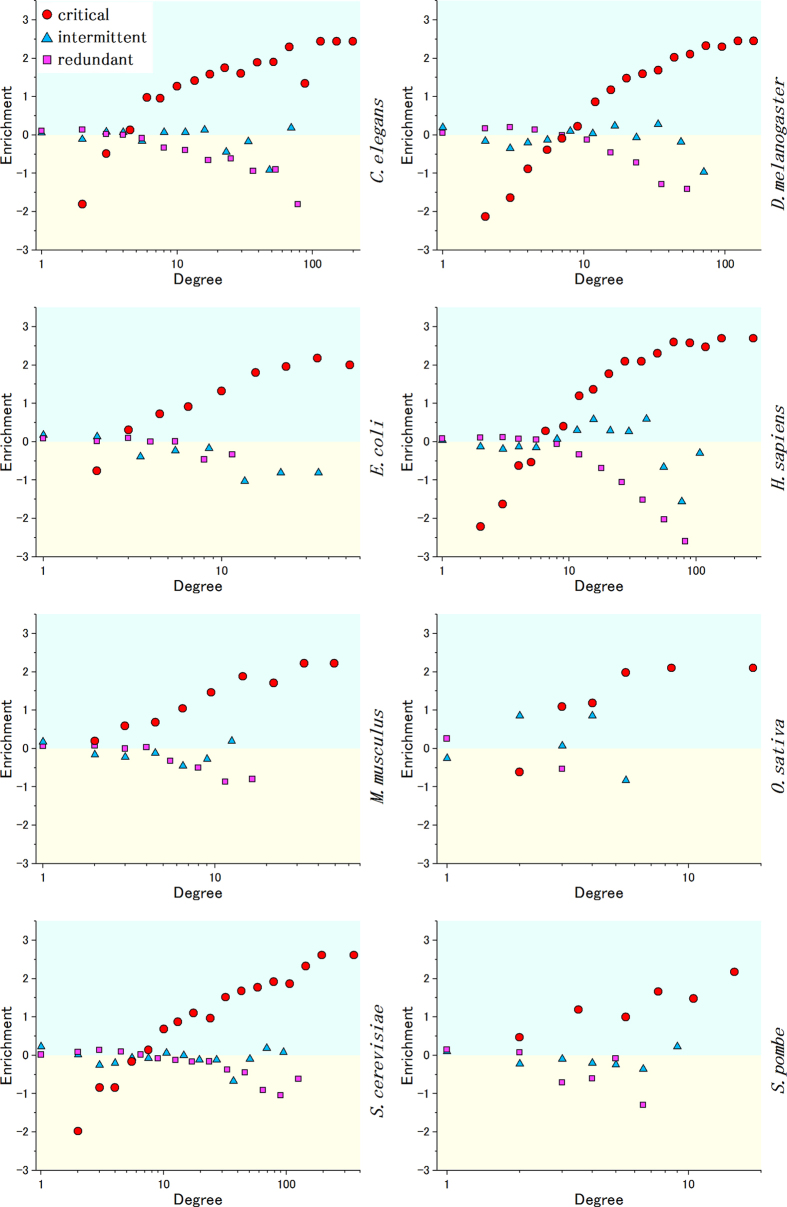
The enrichment of critical, intermittent and redundant nodes versus node degree for each organism. To measure the statistical proportion of proteins engaged in a given control set *S* (critical, intermittent and redundant) according to their degrees, we performed the following enrichment computation, which was also used by Wuchty^9^. First, proteins were classified according to their degree in logarithmic bins of increasing size. For each bin class *i,* we computed the frequency of proteins with degree *k* as 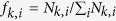. Next, we calculated the fraction of proteins with degree *k* that also appeared in a given set *S* as 
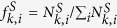
. Then, the enrichment of proteins with degree *k* that appear in the control set *S* (critical, intermittent and redundant) in bin *i* was computed as 
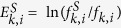
. Positive values of this function indicate the enrichment of degree *k* for the critical, intermittent and redundant set, respectively. Negative values indicate depletion of degree *k*.

Using the newly discovered critical set algorithm, we first evaluated the extent to which the set of critical proteins is enriched with highly connected proteins. The analysis was performed using protein interaction networks corresponding to eight organisms as shown in Fig. 3. The data used to build the networks were obtained from the High-quality INTeractomes (HINT) database V2.0^22^ for all organisms, except for *E. coli*, as the data for this bacterium were collected from ref. 23. The number of proteins and interactions in each organism are summarized in Table S1 (see the Methods section for details). The critical set is systematically enriched for higher-degree nodes. By contrast, the analysis of the set of redundant nodes reveals a depletion of nodes with high degrees and a small enrichment in nodes for low degrees. The remaining nodes are classified as intermittent and accordingly are sometimes engaged in network control. The computational analysis shows that in almost all cases, this set is also moderately enriched when the degree of nodes increases. However, the degree of enrichment is in most cases several times lower than that of the maximum degree observed for critical nodes. The case of the human protein network clearly illustrates the observed general tendency in all organisms. Taken together, high-degree nodes tend to be critical, medium-degree nodes may tend to engage in network control intermittently and low-degree nodes are more associated with redundant and occasionally with intermittent network control roles.

### Proteins engaged in critical network control are also enriched in essential gene products

The finding that the most highly connected proteins in the cell are associated with critical network control poses the question of whether these proteins are also directly required to sustain life. To answer this question, we collected essential genes from the Database of Essential Genes (DEG)^3^. To assess humans, we also compiled data from the Online Gene Essentiality Database (OGEE)^24^. The numbers of essential genes compiled for each organism and the databases from which they were extracted are summarized in Table S2 (see SI). For each organism, we then mapped a gene product for each essential gene to the protein interaction network. To precisely map the gene product onto the proteins, we used the UniProt (Swiss) and UniProt (Swiss & TrEMBL) database annotations. Using the resulting data, we defined a contingency table and evaluated the statistical significance of the association between critical proteins and essential genes by applying Fisher’s exact test. The two-tailed exact p-values, presented in Table 1, showed that optimized proteins engaged in critical control were statistically significantly enriched with essential genes for all analysed networks (*P* < *0.05*) except for that of *S. pombe*, which is not surprising given the few statistics available for this organism, as shown in Fig. S9 and Table S1. The enrichment factors for all the analysed organisms are shown in Fig. S10. In consistency with the p-value analysis, all organisms except for the *S. pombe* exhibit high enrichment factors.

As shown above, one of the main findings of this work is the high association between the critical control node set and the essential gene set. Another is the high association between the critical control genes and the high degree genes. These results raise question of to what extent the critical control accounts for the association with the essentiality, when hubness (degree) is controlled. To address this question, we defined and computed an enrichment factor that captures this dependency. The results for all analyzed organisms are shown in Fig. S11. It is clear from the figure that critical nodes are enriched in essential genes for most degree intervals except very low degree and high degree nodes. It is to be noted that most of high degree nodes are also critical nodes and thus the enrichment factor would be 0 or very close to 0. It should also be noted that the number of essential nodes is small for low degree nodes and thus the enrichment factor is sensitive to small perturbations.

It is also important to note that the analysis of biological data, such as the protein interaction network has always suffered from some research induced by the fact that important gene (such as possibly essential genes) are studied better so they may have more interactions identified than those of less important genes. Therefore, this fact should also be taken into account when interpreting the results. Our study uses data from eight organisms, including *H. sapiens*. In particular, we compiled three different datasets for essential genes in humans to minimize this statistical issue.

### Essential proteins engaged in critical control are also enriched in each Gene Ontology annotation category

**Figure 4:**
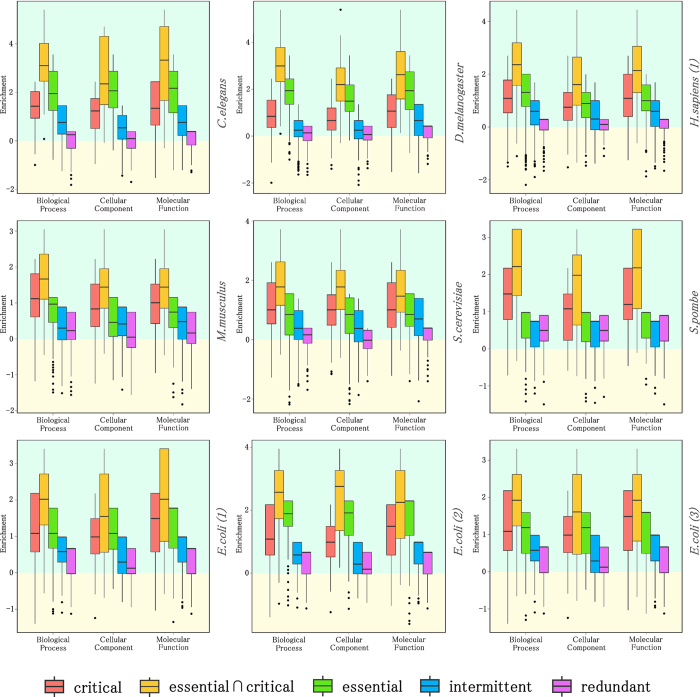
The box-and-whisker plot of the proteins enriched for each Gene Ontology annotations: biological process, molecular function, and cellular component. Each protein was classified into five categories based on whether it is engaged in critical (red), intermittent (blue) or redundant (purple) network control and also on whether it plays an essential (green) or an essential and critical control role simultaneously (orange). The calculation of the enrichment of each category was then calculated as shown in the Methods section. The results show that those essential proteins engaged in critical control (orange) also show the highest enrichment in biological process, molecular function and cellular component annotations. The strongest signal for their associations with GO categories corresponds to *H. sapiens, S. cerevisiae, C. elegans, D. melanogaster and E. coli* organisms. Each organism is indicated in the figure. *E. coli* organisms show results for three different essential gene datasets, respectively. The results for *H. sapiens (1)* are shown in figure. For the rest of datasets for *H. sapiens* we obtained very similar results. The statistics and data sources for the essential genes are shown in Table S2. Additional statistical data is shown in Table 2 in main text and Tables S6 and S7 in SI. See also Supplementary Excel files for additional data.

To further understand the biological associations and implications of the proteins engaged in network control, we compiled all the Gene Ontology (GO) annotations (biological process BP, molecular function MF and cellular component CC) using the UniProt database. Each protein was then associated with its annotated GO terms. Each protein was also classified into one of five categories based on whether it is engaged in critical, intermittent, or redundant network control; represents an essential gene product; or is an essential gene product and simultaneously involved in critical control. The categories for the critical, intermittent and redundant are mutually exclusive. The category for the essential genes can contain genes from any control category. Finally, essential genes that are classified as critical are also examined in a separate category. The enrichment of each category was then calculated as shown in the Methods section. The essential proteins engaged in critical control (orange) also show the highest enrichment in biological process, molecular function and cellular component annotations (see Fig. 4). However, it is unclear which is the second best, essential proteins (green) or proteins engaged in critical control (red), because it largely depends on organisms. In particular, the second best differs even within *E. coli* essential genes datasets. The above mentioned observations suggest that the currently available PPI network data combined with essential genes datasets are useful to derive strong or general tendencies but are not enough to derive detailed or weak signals. The observed enrichment decreases when the network control engagement decreases from critical, intermittent and redundant, the latter showing the lowest values and even depletion scores (Fig. 4). This tendency is universal to all the analysed organisms and suggests evidence of a triad association of lethality, network control and biological functionality.

To further analyse the biological functions associated to the critical control in more detail, we examine each annotated term and functional class inside each GO category. The compiled results are available as supplementary files (see Excel files). We have selected the top-five functional classes for each category BP, MF and CC ordered by the number of critical and essential critical genes. The associated enrichment factors and the two-tailed p-values for the Fisher’s exact test are also displayed. The results are shown in Table 2, which corresponds to the *H. sapiens 1* dataset for essential genes. For the *H. sapiens 2* and *H. sapiens OGEE* datasets see SI (Tables S6 and S7).

**Figure 5:**
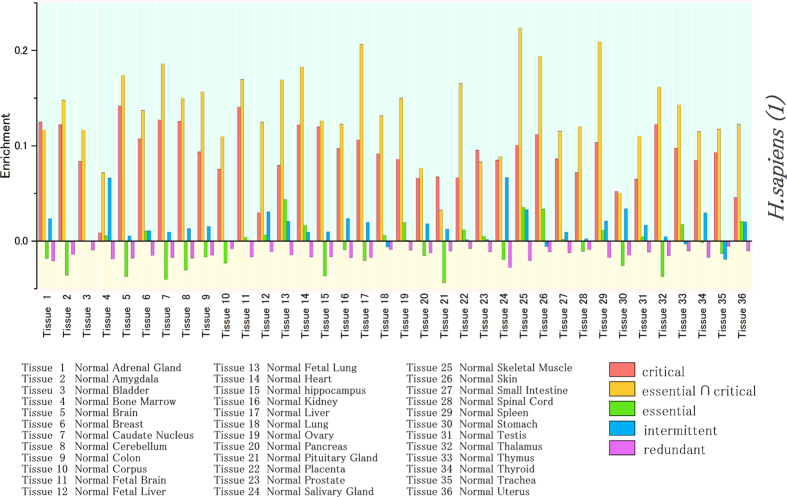
Enrichment of co-expressed genes for each category: critical (red), intermittent (blue), redundant (purple), essential (green) and simultaneously essential and critical control roles (orange) across 36 different healthy human tissues. The subset of co-expressed critical genes that are also annotated as essential genes exhibit the highest enrichment in almost all tissues. The analysis is performed for three different datasets of essential genes as shown in Table S2. The results for *H. sapiens (1)* dataset are shown in figure. The results for the rest of datasets for *H. sapiens* are shown in Fig. S20 in SI.

On the other hand, we have examined data corresponding to functional and evolutionary patterns for the eukaryotic orthologous groups or KOGs^25^,^26^. The results are shown in Fig. S12 and indicate that the essential genes involved in critical control are quite localized and mainly enriched for transcription (K) (*P* = 4.37 × 10^−4^), signal transduction mechanisms (T) (*P* = 5.28 × 10^−5^) and replication (L) (*P* = 6.68 × 10^−2^) functional classes. A similar analysis was done only for MDS in Khuri & Wuchty in Fig. 5 19, although they did not investigate critical control and only examined the category of MDS and MDS with essential genes. Interestingly, a similar set of highly enriched functional classes (K,L,T) were identified in their analysis for the MDS enriched with essential genes. For statistical details related to Fig. S12 see also Supplementary Excel file.

### Dominance of critical co-expressed genes across human tissues

Using the available microarray data from samples of 36 different healthy human tissues^27^, we investigated whether the fraction of co-expressed genes involved in critical control is homogeneously enriched across human tissues. The results shown in Fig. 5 indicate that, although subject to noticeable variations, co-expressed critical genes are enriched in all 36 healthy tissue samples. Moreover, the subset of co-expressed critical genes that are also annotated as essential genes exhibits the highest enrichment across human tissues. By contrast, fractions of redundant co-expressed genes exhibit depletion in almost all human tissues.

**Figure 6:**
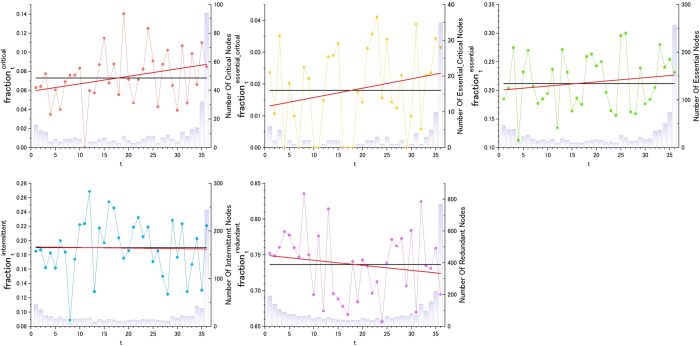
The fraction of genes in a particular set of critical (**a**), essential and critical (**b**), essential (**c**), intermittent (**d**) or redundant (**e**) genes among genes whose transcripts are expressed in *t* tissues, from 1 to 36. For each figure (**a–d**), the scale for the grey bars is marked on the right axis and indicates the number of proteins found in each tissue. The fraction of critical and essential critical proteins increases when the number of tissues in which they are expressed also increases. The essential gene dataset corresponds to *H. sapiens (1)*. The results from two other human essential gene datasets are shown in SI.

The ubiquitous feature of the critical and essential critical nodes can be observed in Fig. 6(a–d). This figure shows the fraction of genes in a particular subset *S* among those whose transcripts are expressed in *t* tissues, from 1 to 36. The fraction of critical and essential critical proteins increases when the number of tissues in which they are expressed also increases. This tendency shows that network control roles also require an optimized subset of housekeeping genes that are present in the majority of tissues. When the fraction of intermittent and redundant genes is examined, the correlation is reverted progressively, indicating that they are not ubiquitous in all tissues.

Although the analysis shown in Fig. 6 indicates that network control roles require a set of genes that are always present (co-expressed) in the majority of tissues, it does not answer the question on the specific evolutionary conserved biological functions associated to each of these genes. To address this question we proceed as follows: First, we have selected all genes associated to critical control (which also includes essential critical genes). We then only extracted from this set, those genes that are expressed in 35 or more different tissues simultaneously (all samples include 36 different healthy tissues). Then, we then mapped the KOG biological functions on the resulting set of genes. Genes present in the KOG set but absent in the compiled PPI network were discarded in the analysis. The gene names as well as the KOG functions are displayed in Table 3 for the *H. sapiens 1* dataset. See SI in Tables S8 and S9 for the rest of datasets. Moreover, the number of critical co-expressed genes in each functional class is visualized using a bar plot in Figs S13 and S14. The Information processing and storage (red) and cellular processes and signalling (blue) are the KOG categories with the highest number of critical genes simultaneously co-expressed in most tissues.

### Linking structural controllability with dynamic co-expression synchronization

**Figure 7:**
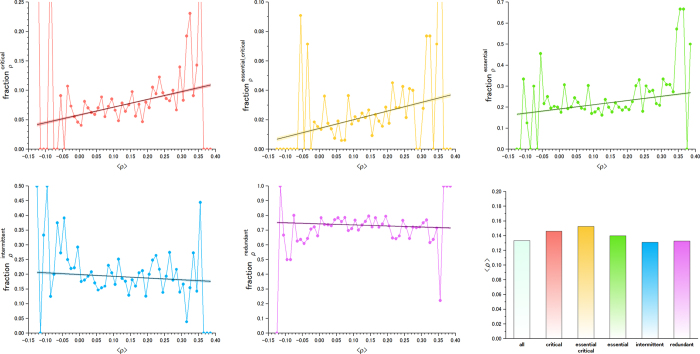
The fraction of genes for each category (critical (**a**), critical and essential (**b**), essential (**c**), intermittent (**d**) and redundant (**e**)) among those whose average Pearson Correlation Coefficient (PCC) with all other genes in the cell is <*ρ*>. See the Methods section for details. (**f**) The PCC averaged for all genes included in each category. The essential gene dataset corresponds to *H. sapiens (1)*. The results for two other human essential gene datasets are shown in SI. The linear fit to the data (black line) also shows the 95% confidence band in the same colour as the data points. When it is too small, it may not be visible. The exact p-values for the analysis of statistical significance analysis are shown in SI, Table S5.

To perform complex functions, genes associated with control roles must coordinate the activity of numerous other genes in a synchronized fashion. Essential genes, for example, have been reported to show an expression pattern that is synchronized with a large number of other genes^28^. Here, we find that the average co-expression coefficient of the optimized set of essential genes involved in critical network control shows the greatest synchronization with all other genes in the cell (see Fig. 7(b,f)). The intermittent and redundant nodes show a lower average correlation with other genes, consistent with what we would expect for a less specialized functional role in cellular control (Fig. 7(f)). This result is important because it captures a direct link between the structural controllability of genes and dynamic transcriptional synchronization features for the first time.

## Discussion

In this work, we have presented an efficient computation of optimized critical control and redundant control subsets in scale-free networks that drastically surpasses existing algorithms; the computational time is reduced by as much as 180 times, and the network size for application is expanded by as much as 25 times, from 1,000 to 25,000 nodes in the best cases. Because most real-world networks follow a scale-free topology, in particularly the protein networks discussed in this work, the proposed algorithm may potentially lead to multiple applications in various fields.

The developed algorithmic procedure allowed a detailed examination of the control categories of proteins and their relationships with human orthologues of mouse essential genes in proteome-wide protein interaction networks for eight organisms. The optimized subset of proteins involved in critical control was found to be enriched for the most highly connected proteins and largely depleted of less connected ones. Proteins engaged in critical network control were also enriched in essential gene products. The optimized subset of essential proteins engaged in critical control was also enriched in each Gene Ontology annotation category. This observed tendency is universal to all the analysed organisms and suggests evidence of a triad association of lethality, network control and biological functionality. The analysis of transcriptome-wide gene expression profiling corresponding to 36 types of normal human tissues also produced a novel observation correlating structural controllability properties and dynamic transcription features. The fraction of genes associated with critical roles and their overlap with essential genes increased when the number of tissues in which they are expressed also increased. This tendency showed that control roles also require the presence of an optimized subset of housekeeping genes in the majority of tissues. Moreover, our findings revealed that the average co-expression coefficient of the optimized subset of essential genes associated with critical control showed the highest synchronization with all other genes in the cell, capturing a direct link between structural controllability of genes, the gene’s lethality and the synchronization of dynamic co-expression for the first time. We believe that the identified optimized critical network control subsets may be of interest as drug targets; thus, they may be useful in drug design and development.

## Methods

### Protein Interaction Database

The data corresponding to protein-protein interactions for the organisms shown in Table 1 were compiled from the High-quality INTeractomes (HINT) database^22^ using binary interactomes for all organisms. The protein interactions for *H. sapiens* correspond to the high-throughput interactome. In addition, the interactions corresponding to *E. coli* were collected from a separate data source^23^.

### Databases of Essential Genes

The essential genes for the analysed organisms were compiled from the Database of Essential Genes (DEG) database^3^. For humans, we considered three datasets in our study. Two of them are accessible from the DEG: the results from Liao and Zhang in^29^, with 118 genes, and the results from Georgi *et al.* in^5^, with 2,452 genes. The latter dataset is referred to in the results as *H. sapiens 1*, and the two datasets together form *H. sapiens 2.* These genes represent human orthologues of mouse essential genes, which are associated with 46 phenotypic categories with prenatal, perinatal and postnatal lethality. An additional dataset from OGEE (Online Gene Essentiality Database) was also included in the analysis^24^, and it is referred to as *H. sapiens* (*OGEE*). The numbers of essential genes compiled for each organism are summarized in Table S2.

### UniProt (Swiss and Swiss & TrEMBL) Database

The annotations for the proteins were retrieved from the UniProt database. We used both the Swiss and Swiss-TrEMBL repositories for the mapping of essential genes on proteins. These are used to compute the two-tailed P-values using Fisher’s exact test (see Table 1). The Gene Ontology annotations for biological processes, molecular function and cellular component were also compiled from the UniProt database.

### Enrichment calculation for each Gene Ontology annotation: Biological process, molecular function, and cellular component

The enriched proteins in a given Gene Ontology (GO) annotation that also appear in the critical set were determined as follows. First, we calculated the fraction of the number of proteins associated with a GO term *i* (*N*_*GO*(*i*)_) in the entire network of size *N* as *f*_*GO*(*i*)_ = *N*_*GO*(*i*)_/*N*. Next, the fraction of the number of proteins associated with a term *GO(i)* that appeared in the critical set (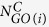) of size *N*^*C*^was calculated as 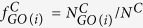. Then, the enrichment of proteins in a *GO(i)* for the critical set *C* was computed as 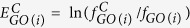. Next, each value for enrichment was classified into one of three groups according to whether its GO term was related to biological process, molecular function or cellular component. Finally, for all the enrichment values 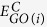 corresponding to the GO terms in each annotation class, a box-and-whisker plot was constructed. In each box, the bottom and top indicate the first *q*_*1*_ and third *q*_*3*_ quartiles (the 25^th^ and 75^th^ percentiles, also called hinges), and the bold blue band inside the box represents the second quartile (the median). The upper (lower) whisker extends from the hinge to the highest (lowest) value that is within 1.5 *x* IQR of the hinge, where IQR is the inter-quantile range or distance between *q*_*1*_ and *q*_*3*_. Data not included between the upper and lower whiskers are plotted with dots (also called outliers). The separations between the different parts of the box-plot denote the degree of dispersion (spread) in the data set.

### Gene expression data and analysis

In our analysis, we used data from genome-wide expression profiling corresponding to 36 types of normal human tissues^27^. The number of genes in a particular subset *S* (essential, critical, critical and essential, intermittent and redundant) with an average gene co-expression coefficient <*ρ*> is denoted as 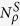. The total number of genes with an average gene co-expression coefficient <*ρ*> is *N*_*ρ*_. Then, the fraction of genes in a particular subset *S*, among those whose average gene co-expression coefficient with other genes is <*ρ*>, reads as 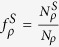. This measure is used in Fig. 7 (vertical axis). The average gene co-expression coefficient of gene *i* with respect to all other genes *j* reads as 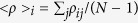, where *ρ*_*ij*_ is the Pearson Correlation Coefficient (PCC) between genes *i* and *j.*<*ρ*>_*i*_ is presented in Fig. 7 (horizontal axis), where index *i* is dropped for readability purposes.

Two key procedures are performed to process the data, which correspond to the analogous protocol used in^28^.

1. Microarray Data Normalization: The raw gene expression data for 36 tissues^27^ were first normalized to mean-centre for different tissues. The normalization factor was obtained for each tissue by summing up the data for the probes that are “*present*” (significant p-value) in all 36 tissues. The term present refers to genes with an expression level that has sufficient statistical significance (P-value < 0.01).
2. Handling multi-probe genes: Some genes are tagged by multiple probes in the data from Ge *et al.*^27^. To assign a single value of gene expression correlation, we took the best (largest) Pearson correlation coefficient among possible pairs of two gene probes to capture the best possible coordinated activities of the two genes among different tissues. The PCC was calculated with the log (normalized data). The probes that are “*absent*” (no significant p-value) in all 36 tissues were discarded from the analysis. For a few cases of probes mapped to multiple gene annotations, we simply prioritized the annotation for the largest expression value observed (the more reliable observation) and its role category, for example, essential and critical.

### An algorithmic procedure for efficiently computing optimized critical and redundant subsets in scale-free networks

Nacher and Akutsu presented an algorithm to compute the optimal critical and redundant sets of nodes^15^. The algorithm, although providing the optimal and exact solution using ILP, needs to solve the MDS problem *n* times, where *n* is the number of nodes (see Supplementary Information (SI) for details). Therefore, if compared with the computation of a single MDS, the method requires much more CPU time, and the feasible size of networks is limited to approximately 1,000 nodes. This severe limitation is problematic in the analysis of critical network control features in many real-world networks, including cellular networks. To by-pass the strong computational requirements of the problem, we designed an algorithm that exploits the scale-freeness property observed in most of the real-world networks. First, the mathematical analysis presented by Nacher and Akutsu^15^ derived an important proposition related to the nodes engaged in critical control:

#### Proposition 2.2 (Critical proposition)

If node *v* has two or more neighbouring nodes with degree *k* = 1, *v* is a *critical node*^15^.

Here, we present a new proposition to identify nodes engaged in a redundant control role.

#### Proposition 1 (Redundant proposition)

If all neighbours of a node *v* are critical nodes, *v* is a *redundant node*.

Using the above two propositions and taking advantage of the heterogeneous degree distribution observed in scale-free networks, we can substantially enlarge the computable size of networks. The degree distribution *P(k)* of a scale-free network for nodes of degree *k* follows a power law with *k*^−*γ*^ functional form, where *γ* indicates the degree exponent of the network. This means that most of the nodes have a low degree; many of them are degree *k* = *1*, and only a small fraction of nodes are highly connected. Therefore, using proposition 2.2^15^, (1) we can efficiently pre-compute an optimal subset of critical nodes. This allows us to substantially reduce the number of times we need to solve ILPs. In addition, (2) because the number of constraints, in addition to the trivial ones, is significantly smaller, the ILP can solve each ILP faster and therefore provide optimal and exact solutions for larger networks (see SI for details).

The algorithm for computing the optimized set of redundant nodes benefits from Proposition 1. Because highly connected nodes tend to be critical nodes, there are many low-degree nodes connected only to critical nodes. Therefore, (1) by direct application of proposition 1, a large number of redundant nodes can be pre-determined before computing the ILPs. As in the critical set case, (2) the number of constraints to be solved in the model for each ILP is drastically reduced, which allows faster computations in larger networks.

Taken together, our results show that a simple procedure surprisingly pre-determines a large number of critical (*c*) and redundant (*r*) nodes *m* = *c* + *r* in a network of size *n.* Then, the role of the remaining unassigned nodes *(n-m*) can be determined using the algorithm proposed by Nacher and Akutsu^15^, which only requires (*n-m*) ILP computations with lower constraint complexity.

### Algorithm description

Here, we summarize the new algorithmic procedure which uses a novel pre-processing step for significant speed-up computation. A detailed example of the computation is shown in SI.

1. We apply the **Critical proposition** for each node, which states that if node *v* has two or more neighboring nodes with degree *k* = 1, *v* is a critical node.
2. We apply the **Redundant proposition** for each remaining node, which states that if all neighbors of a node *v* are critical nodes, *v* is a redundant node. Note that from 1 and 2 pre-processing steps, a preliminary set of critical and redundant nodes are identified together a lower bound |*M*_*L*_| of the MDS, without any ILP computation.
3. We apply the algorithmic procedure proposed by Nacher and Akutsu in^15^ to determine the control category of the remaining nodes.

3.1. We first solve the simplified MDS problem for the network after adding information obtained from 1 and 2 steps into the ILP.

3.2. Apply the critical algorithmic procedure^15^ to each node that belongs to MDS, and is not already identified as critical, to determine its critical role.

3.3. Apply the redundant algorithmic procedure^15^ to each node that does not belong to the MDS to determine its redundant role.

## Additional Information

**How to cite this article**: Ishitsuka, M. *et al.* Critical controllability in proteome-wide protein interaction network integrating transcriptome. *Sci. Rep.* **6**, 23541; doi: 10.1038/srep23541 (2016).

## Supplementary Material

Supplementary Information

Supplementary Dataset 1

Supplementary Dataset 2

Supplementary Dataset 3

Supplementary Dataset 4

Supplementary Dataset 5

Supplementary Dataset 6

Supplementary Dataset 7

## Figures and Tables

**Table 1:** The two-tailed p-values for the Fisher’s exact test show that optimized proteins engaged in critical control are statistically significantly enriched with essential genes for all analysed networks (*P* < *0.05*) except for that of *S. pombe*, which is not surprising because of the few collected statistics for this organism, as shown in Fig. S9 and Table S1.

Organism	P-value (Swiss)	P-value (Swiss & TrEMBL)
*C. elegans*	2.51E-01	1.55E-02
*D. melanogaster*	5.67E-03	3.12E-03
*H. sapiens 1*	1.87E-02	1.86E-02
*H. sapiens 2*	1.93E-02	1.92E-02
*H. sapiens OGEE*	1.96E-03	1.94E-03
*M. musculus*	7.83E-03	7.10E-03
*S. cerevisiae*	3.01E-07	3.01E-07
*S. pombe*	8.63E-01	8.63E-01
*E. coli 1*	1.41E-04	1.41E-04
*E. coli 2*	9.20E-03	9.20E-03
*E. coli 3*	4.25E-04	4.25E-04

The *C. elegans* results show statistical significance (*P* *<* *0.05*) for the Swiss & TrEMBL dataset. For *E.coli* MG1655, we also compiled several datasets. *E. coli* 1 and 2 refer to data from Gerdes *et al.*^30^ and Baba *et al.*^31^, respectively. Both datasets were available in the DEG. *E. coli* 3 refers to the combined data from *E. coli* 1 and *E. coli* 2. For *H. sapiens* datasets see details in methods section.

**Table 2:** The top-five Gene Ontology (GO) terms for the three GO categories ordered by the number of critical and essential critical genes.

GO category	Go term	Number of		Enrichment		p-Value	
		critical	essential critical	critical	essential critical	critical	essential critical
Biological Process	transcription, DNA-templated	67	15	0.29	0.53	1.27E-02	4.40E-02
Biological Process	gene expression	52	13	0.42	0.78	1.59E-03	7.52E-03
Biological Process	positive regulation of transcription from RNA polymerase II promoter	51	21	0.59	1.45	2.71E-05	6.78E-09
Biological Process	innate immune response	43	14	0.72	1.34	3.81E-06	1.17E-05
Biological Process	viral process	41	9	0.58	0.81	2.06E-04	1.76E-02
Cellular Component	cytoplasm	208	45	0.35	0.56	2.62E-10	6.19E-06
Cellular Component	nucleus	198	47	0.24	0.54	1.91E-05	4.69E-06
Cellular Component	cytosol	155	37	0.41	0.72	7.75E-09	1.90E-06
Cellular Component	nucleoplasm	128	36	0.26	0.74	6.77E-04	2.28E-06
Cellular Component	plasma membrane	87	20	0.24	0.52	1.14E-02	1.60E-02
Molecular Function	ATP binding	63	16	0.38	0.76	1.46E-03	2.89E-03
Molecular Function	identical protein binding	39	10	0.89	1.27	1.83E-07	4.38E-04
Molecular Function	sequence-specific DNA binding transcription factor activity	39	13	0.36	1.01	1.78E-02	7.24E-04
Molecular Function	ubiquitin protein ligase binding	33	9	1.26	1.70	8.77E-11	3.27E-05
Molecular Function	ligase activity	33	5	0.83	0.69	5.06E-06	1.06E-01

The associated enrichment factors and the two-tailed p-values for the Fisher’s exact test are also displayed. The result corresponds to the *H. sapiens 1* dataset. For the *H. sapiens 2* and *H. sapiens OGEE* datasets see Supplementary Information.

**Table 3:** The list of critical genes that are co-expressed in 35 or more human tissues classified according to the KOG functional class for the *H. sapiens 1* dataset.

KOG Functional class	Function	Number of critical genes	Gene name
A	RNA processing and modification	5	DDX24, DDX39B, U2AF2, SF3B3, UPF3A
B	Chromatin structure and dynamics	1	TLE1
C	Energy production and conversion	1	ATP6AP1
D	Cell cycle control, cell division, chromosome partitioning	2	MOB4, PEA15
E	Amino acid transport and metabolism	1	GOT2
F	Nucleotide transport and metabolism	0	
G	Carbohydrate transport and metabolism	1	GAPDH
H	Coenzyme transport and metabolism	1	ALAS1
I	Lipid transport and metabolism	1	FDFT1
J	Translation, ribosomal structure and biogenesis	4	EEF1A1, RPLP1, RPL8, RPS3A
K	Transcription	9	XBP1, CTBP1, MAX, TCF4, ZNHIT3, TCF12, SKP1, MED23, TCEB1
L	Replication, recombination and repair	1	XRCC6
M	Cell wall/membrane/envelope biogenesis	0	
N	Cell motility	0	
O	Posttranslational modification, protein turnover, chaperones	14	UBE2I, UBE2D3, UBE2D2, UBE2D4, RNF11, UBE2E1, UBE2E3, UBE2N, UBE2K, YWHAE, UBE3A, UBE2L6, DNAJA1, CDC34
P	Inorganic ion transport and metabolism	1	SAT1
Q	Secondary metabolites biosynthesis, transport and catabolism	0	
R	General function prediction only	8	EWSR1, CDC42, PLEKHF2, RAC1, LMO4, RAP2A, KLF10, PPFIA1
S	Function unknown	1	WBP11
T	Signal transduction mechanisms	13	NCK1, FYN, CRK, PIK3R1, ACVR1, MAPK14, NUDT3, ARHGDIA, PSEN1, MPP3, PRKAR1A, MAPK10, PPP2CA
U	Intracellular trafficking, secretion, and vesicular transport	1	SEC23B
V	Defense mechanisms	0	
W	Extracellular structures	0	
Y	Nuclear structure	1	NSFL1C
Z	Cytoskeleton	7	MAP1LC3B, NDEL1, TUBGCP4, PFN2, GABARAPL2, ARPC3, DYNLL1

See SI for the rest of datasets.
