# Supplementary material for: Critical controllability in proteome-wide protein interaction network integrating transcriptome
Source: Sci Rep. 2016 Apr 4;6:23541. doi: 10.1038/srep23541 (PMC4819195; doi:10.1038/srep23541)
Supplement: Supplementary Information [file srep23541-s1.doc]

**-Supplementary Information-**

**Critical controllability in proteome-wide protein interaction network integrating transcriptome**

Masayuki Ishitsuka^1^, Tatsuya Akutsu^2^ and Jose C. Nacher^1^*,

**Details on the algorithmic procedure for efficiently computing optimized subsets of critical and redundant nodes in scale-free networks.**

In order to compute an MDS, we use an integer linear programming (ILP)-formulation, in which $x_{v}$ is an integer variable associated to a node$v$, and $x_{v}=1$ (respectively, $x_{v}=0$) denotes that $v$ is in the MDS (respectively $v$ is not in the MDS). Let us consider the following network $G(V,E)$with *|V|*=13 nodes and *|E|*=12 edges and apply the following ILP for solving the MDS.


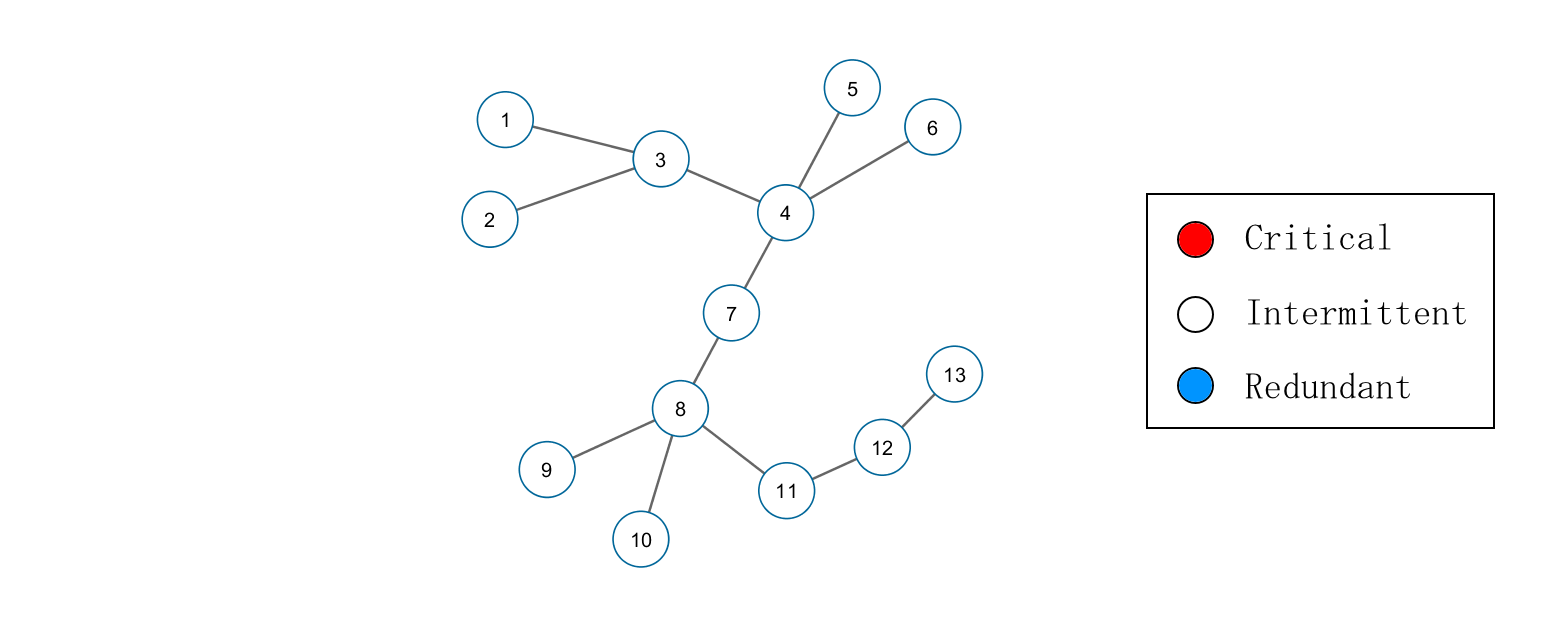


**Fig. S1**: An example of a network of *N*=13 nodes in which the algorithm computes critical, intermittent and redundant sets.

**minimize**

$$\sum_{v\in V} x_{v}=x_{1}+x_{2}+x_{3}+x_{4}+x_{5}+x_{6}+x_{7}+x_{8}+x_{9}+x_{10}+x_{11}+x_{12}+x_{13}$$

**subject to**

$x_{v}+\sum_{(u,v)\in E} x_{u}\geq1$ $\forall v\in V,$ Eq. 1

$x_{v}\in\{0,1\}$ $\forall v\in V$.

It is clear for the above expression that to identify and minimize the MDS, we need 13 equations (one for each node) that constrain the possible solutions. The MDS is then obtained via ILP which is solved one time. In the example, it leads to the following solution: MDS = {3 , 4 , 8 , 12}, so the size of the MDS is *|M*| = 4.

Then, we can compute the number of redundant nodes and critical nodes in the same network. By following Ref. [15] in main text, we can write below the computational procedure to determine de critical set of nodes:

1. Compute an MDS *M* for a graph $G(V,E)$using ILP
2. Let $C_{MDS}$be an empty set.
3. Repeat steps 4-6 for all $v\in M.$
4. Make an ILP instance $I_{v}$ by adding a constraint of $x_{v}\leq0$to the instance given by Eq. 1
5. Get a solution of $I_{v}$ and let $M_{v}=\{v|x_{v}=1\}.$
6. If $I_{v}=\perp$ (i.e. no feasible solution) or , then $C_{MDS}\leftarrow C_{MDS}\cup\{v\}.$
7. Return $C_{MDS}$.

We can see that we have to solve the ILP problem *|M|* times, excluding the computation for the MDS of the original graph and each time we have 14 constrains.

Similarly, the set of redundant nodes can be calculated using the following procedure:

1. Compute an MDS *M* for a graph $G(V,E)$using ILP
2. Let $R_{MDS}$be an empty set.
3. Repeat steps 4-6 for all $v\in V-M.$
4. Make an ILP instance $I_{v}$ by adding a constraint of $x_{v}\geq0$ to the instance given by Eq. 1
5. Get a solution of $I_{v}$ and let $M_{v}=\{v|x_{v}=1\}.$
6. If $I_{v}=\perp$ (i.e. no feasible solution) or , then $R_{MDS}\leftarrow R_{MDS}\cup\{v\}.$
7. Return $R_{MDS}$.

We can see that to compute the set of redundant nodes we need to solve the ILP $|V|-|M|$ times. Therefore, the computation of critical and redundant set of nodes in a network of size *|V| = n,* requires to solve ILPs *n* times, in addition to the original computation for the MDS of size *|M |,* in the original network. Each ILP computation for the critical and redundant sets needs to solve 14 constraints and only one of them is simple as $x_{v}\leq0$ or $x_{v}\geq1$, which corresponds to the tested node.


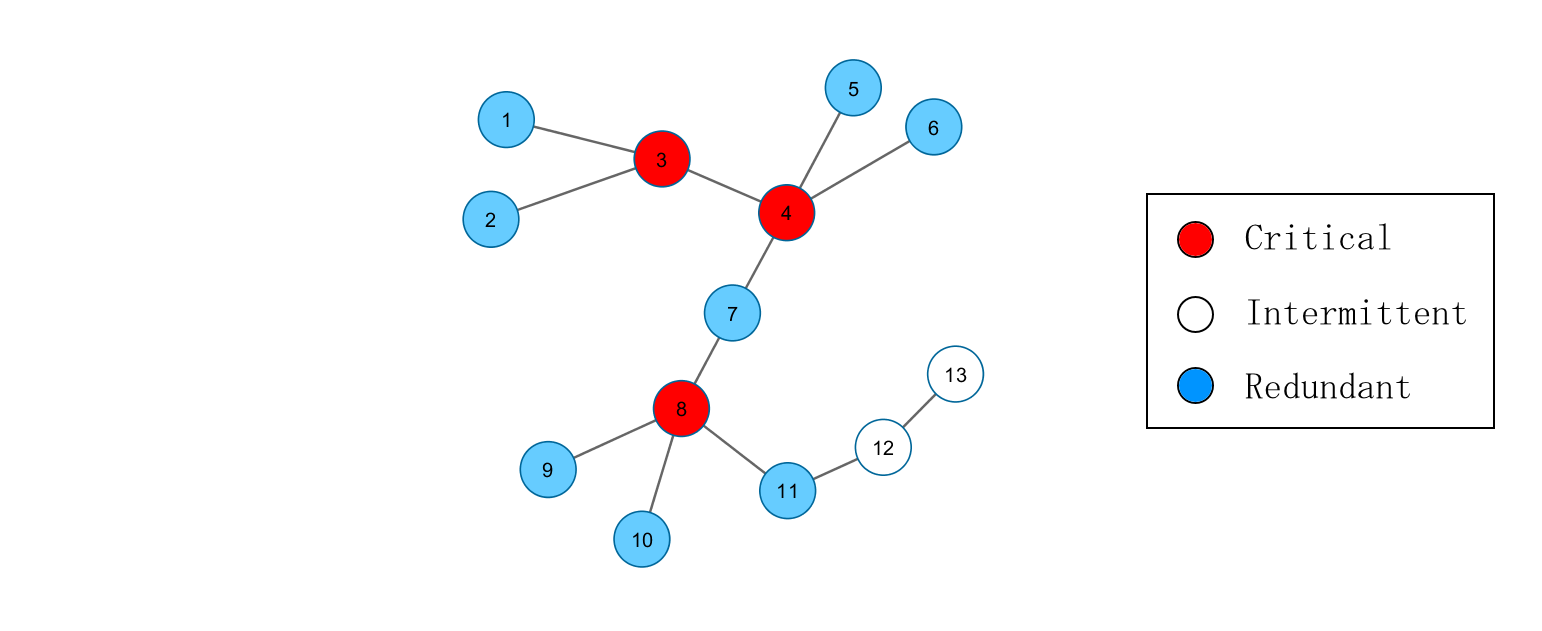


**Fig.S2**: The figure shows the result of the computation of the critical, intermitted and redundant nodes as shown in figure legend.

By following the above procedures we can determine the critical (red), redundant ( blue) and intermittent (white) nodes.

However, when the network is too large the computation of *n* ILPs with complex constraints may not be feasible and the ILP cannot find the optimal solution. To address this computational issue, we consider two propositions refereed as *proposition 2.2* for critical nodes and *proposition 1* for redundant nodes shown in main text, methods section.

Here, we describe the pre-processing step for significant speed-up. We apply the proposition 2.2, which states that if node $v$ has two or more neighboring nodes with degree *k*=1, $v$ is a critical node. Based on this theoretical result (see the proof in ref. [15]), we can automatically identify three critical nodes (red nodes in figure) in the network.

**
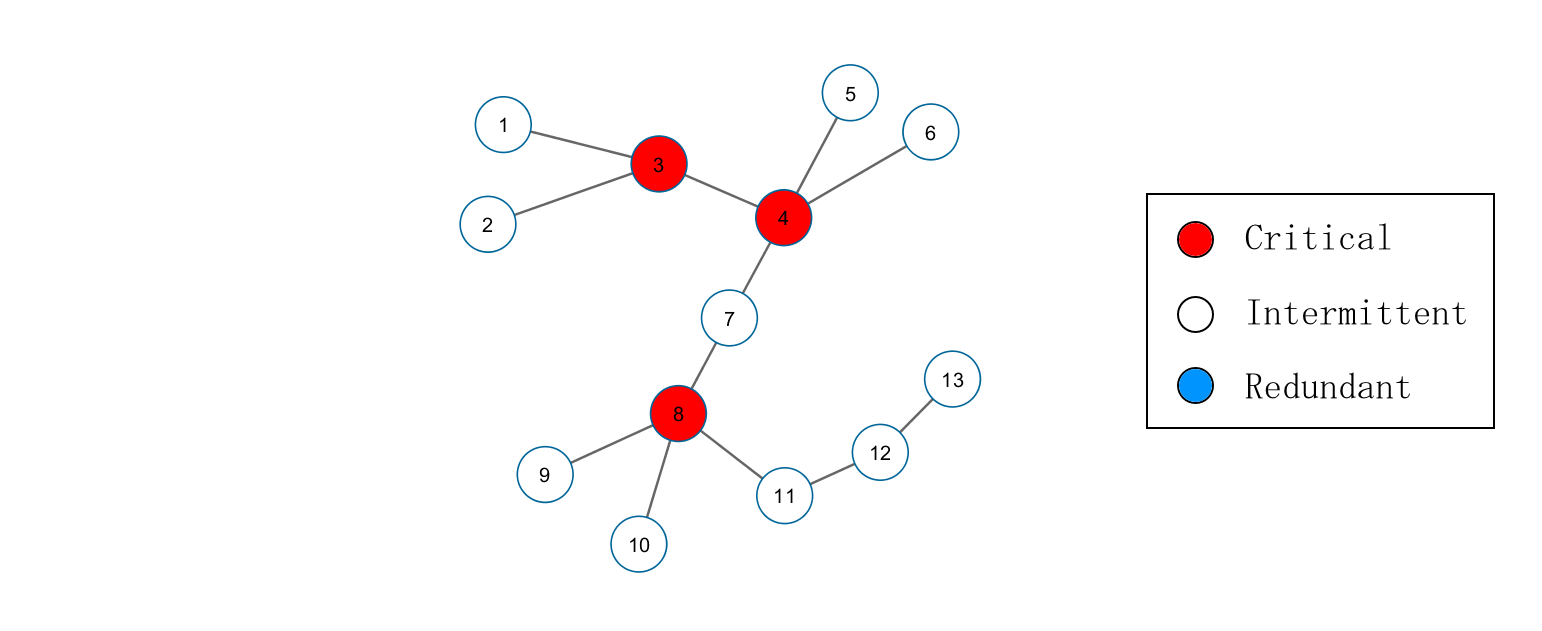
**

**Fig.S3**: The first pre-processing step for critical nodes. The result of the application of proposition 2.2 aids to pre-determine three critical nodes.

Because if they are critical nodes, they must belong to the MDS, the MDS consists at least of nodes {3, 4, 8}. Note that until now we have not solved any ILP yet.

The second processing step requires the application of the proposition 1 for determining a subset of redundant nodes. This proposition states that if all neighbors of a node $v$ are critical nodes, $v$ is a redundant node. Then, we can automatically identify seven nodes (blue) as redundant nodes. As shown in figure, only three nodes remain unidentified after these two processing steps. In addition, we have identified a lower bound $|M_{L}|=3$ of the MDS, without any ILP computation.

**
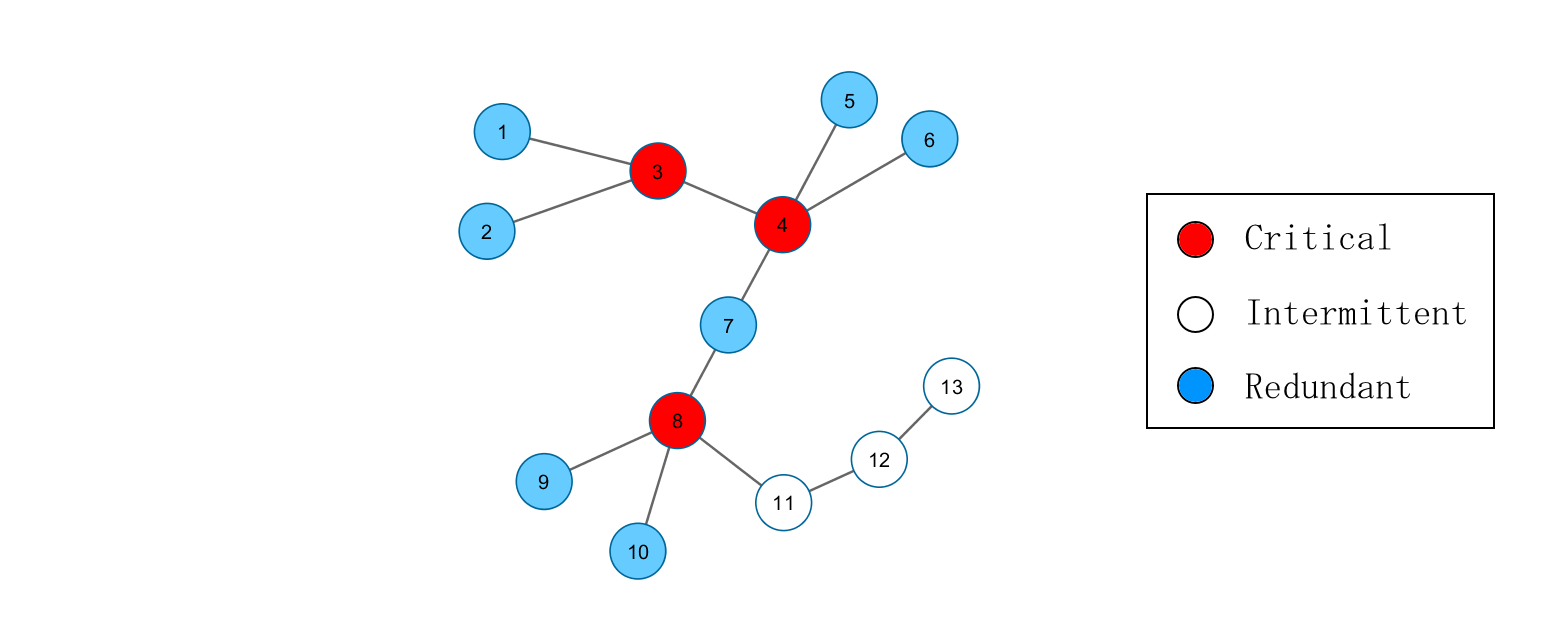
**

**Fig.S4**: The second pre-processing step for redundant nodes. The result of the application of proposition 1 aids to pre-determine seven redundant nodes.

To classify the remaining three nodes (numbering 11, 12 and 13 in white color), we need to apply the algorithmic procedure proposed by Nacher and Akutsu in [15] and summarized above. First, as the procedure states, we need to solve the MDS for the entire network but the computation is notably simplified by replacing $x_{v}=1$ and $x_{v}=0$ for the already identified critical and redundant nodes into Eq.1. We then obtain the following equations:

**minimize**

$$\sum_{v\in V} x_{v}=3+x_{11}+x_{12}+x_{13}$$

**Subject to**

$$x_{11}+x_{12}+x_{13}\geq1$$

$x_{12}+x_{13}\geq1$ Eq. 2

$$x_{v}\in\{0,1\}$$

$$\forall v\in V.$$

By solving this ILP problem, we obtain the MDS = {3 , 4 , 8 , 12}, with size |*M*| = 4. Note that Eq. 2 is notably simpler than Eq. 1. Then, we only need to solve the ILP three more times, each one for each unassigned node. (t=1) We first eva;ute the nodes that remain unassigned in an MDS. The node 12 does belong to the MDS, therefore we apply the critical set procedure by setting the following ILP:

**minimize**

$$\sum_{v\in V} x_{v}=3+x_{11}+x_{12}+x_{13}$$

**Subject to**

$$x_{11}+x_{12}+x_{13}\geq1$$

$x_{12}+x_{13}\geq1$ Eq. 3

$$x_{12}=0$$

where we have added the last contraint to verify its critical role. The solution of this ILP leads to an MDS of size $|M_{12}|=4=|M|$, therefore, the node 12 can only be intermittent.

**
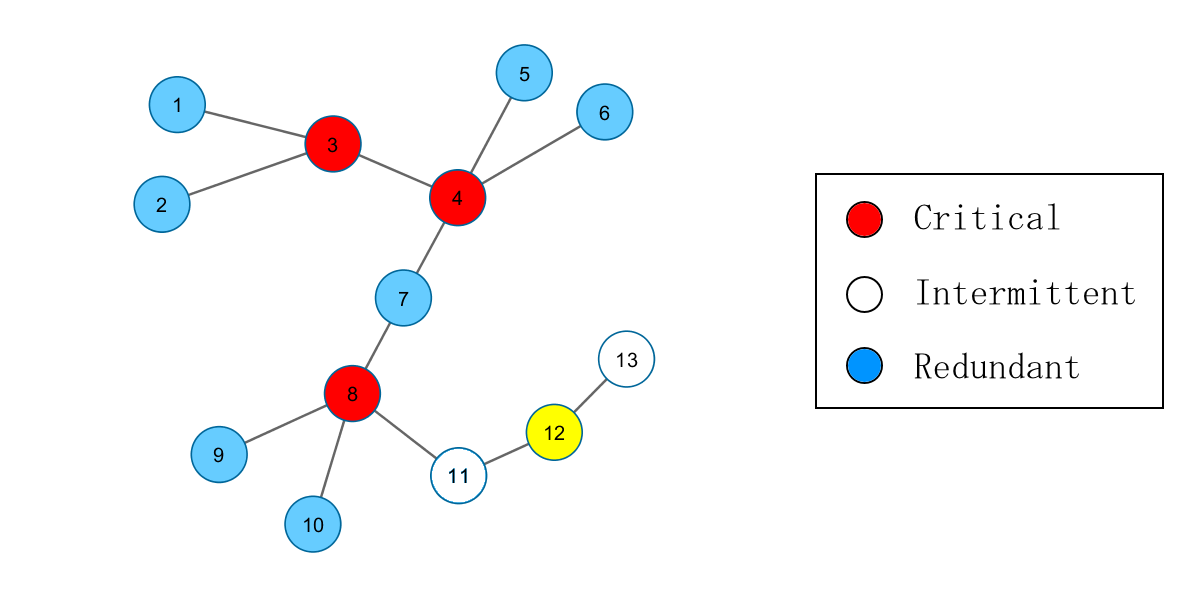
**

**Fig.S5**: The computation of the MDS for the entire network reveals that the node 12 (yellow node) is the only MDS node that is not yet assigned to critical role. We then first verify whether this node is critical or intermitted node using the critical set algorithmic procedure.

Next, we can apply the redundant set procedure for node 11 because it does not belong to the MDS (t=2). Then, we can set the ILP as follows:

**minimize**

$$\sum_{v\in V} x_{v}=3+x_{11}+x_{12}+x_{13}$$

**Subject to**

$$x_{11}+x_{12}+x_{13}\geq1$$

$x_{12}+x_{13}\geq1$ Eq. 4

$$x_{11}=1$$

where we have added the last constraint, because the node does not belong to the MDS and we need to verify its redundant role. The solution of the ILP gives an MDS of size $|M_{11}|=5>|M|$, which indicates the the node 11 is redundant.

**
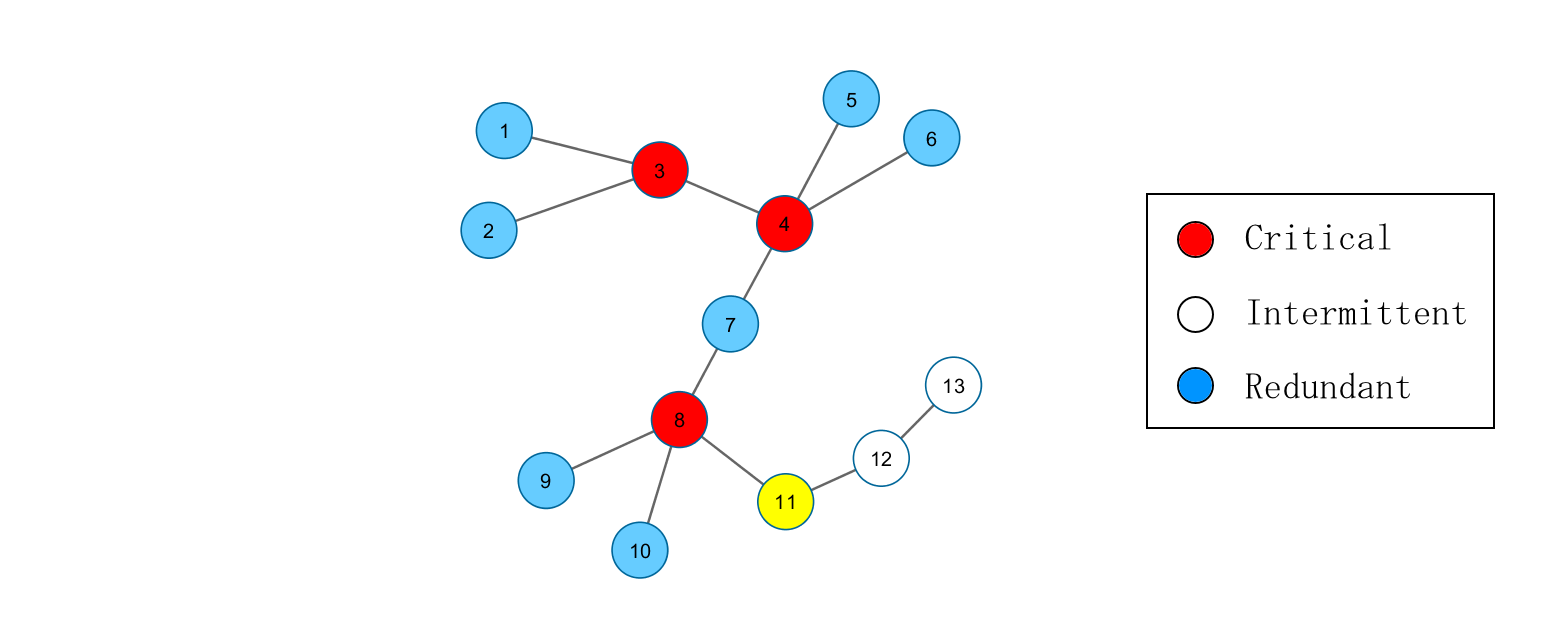
**

**Fig.S6**: Here we examine whether node 11 (yellow) is redundant node using the redundant set algorithmic procedure.

The last node 13 is considered (t=3). This node does not belong to the MDS, therefore the redundant set procedure is applied as follows:

**Minimize**

$$\sum_{v\in V} x_{v}=3+x_{12}+x_{13}$$

**subject to**

$x_{12}+x_{13}\geq1$ Eq.5

$$x_{13}=1$$

where we added the last constraint to verify its redundant role. The solution of this ILP leads to an MDS of size $|M_{13}|=4=|M|$, therefore, the node 13 can only be intermittent.


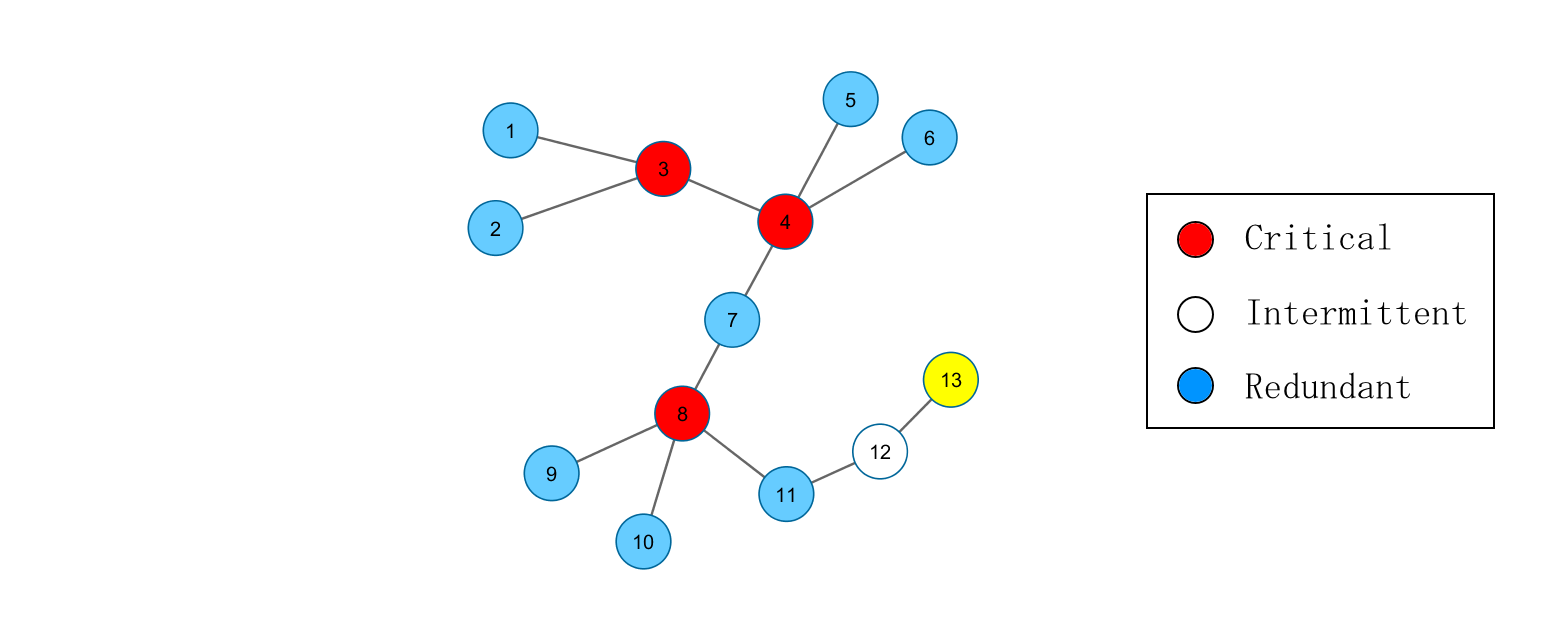


**Fig.S7**: Here we examine whether node 13 (yellow) is redundant node using the redundant set algorithmic procedure.

**Summary**

We summarize here the complexity of the computation. The following two features are essential in decreasing the complexity: (1) By using algorithmic procedures for determining the critical and redundant sets shown in Ref. [15] (main text), we had to solve 13 times the ILPs (plus one more for determining the MDS of the original network) in our network example. In constrast, by using the novel propositions that automatically pre-determine a subset of critical and redundant nodes, we had to solve the ILPs only 3 times (plus one more for determining the MDS after redundant and critical nodes are pre-computed). (2) In addition, the ILPs solved using the newly proposed method have a significantly smaller number of constraints (equations), besides the trivial ones (compare Eq.1 with Eqs. 2-5). These two features seem to be crucial to decrease the computational time and to determine critical and redudant sets in large networks. The relations with network structure of the propositions 1 and proposition 2.2 suggest that scale-free networks have the ideal structure in which the proposed algorithm can be computed most efficiently, as explained in the main text, methods section. Because most of real-world networks follow a scale-free topology, the proposed algorithm may potentially lead to multiple applications in different fields.

**Additional Figures and Tables.**

**
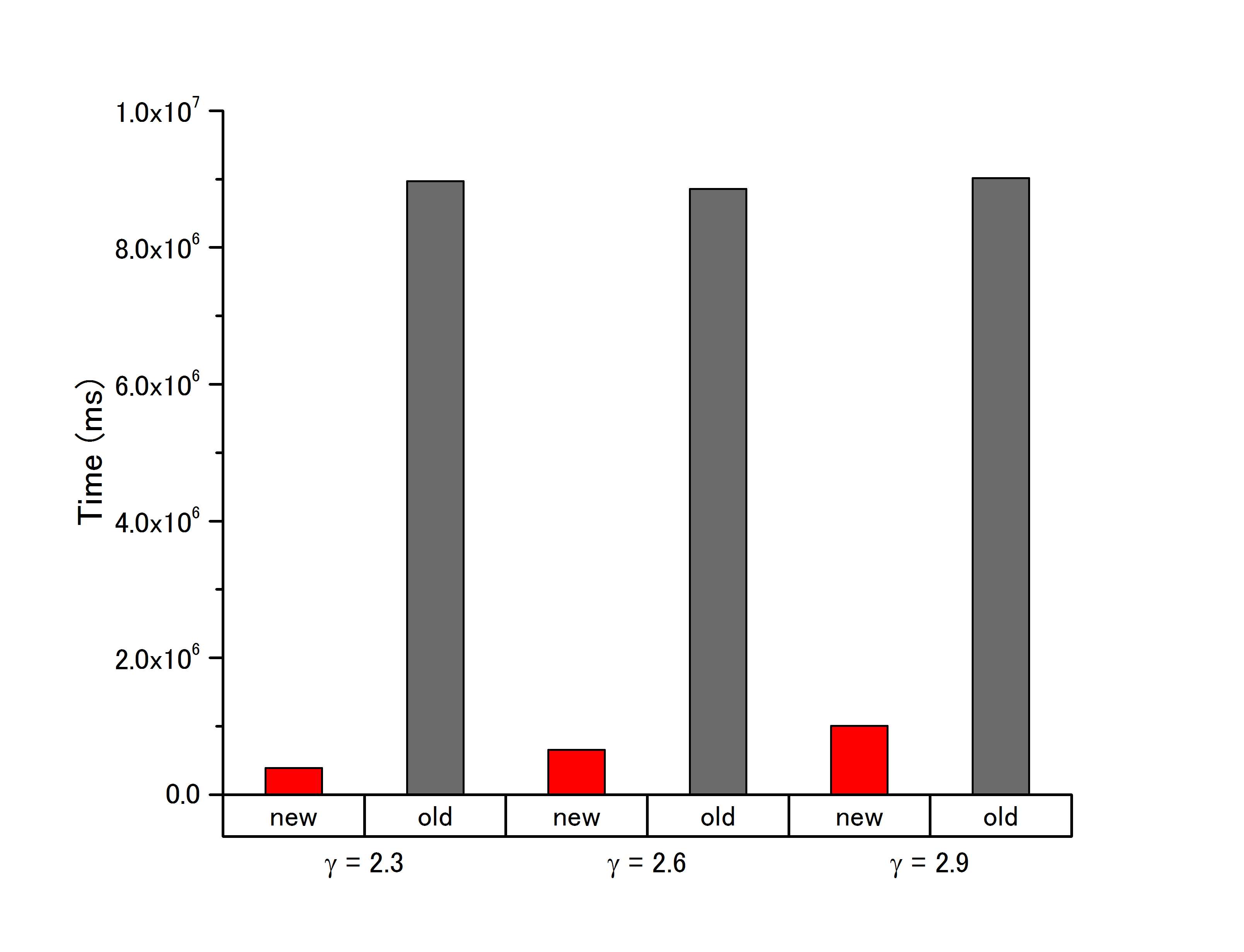
**

**Fig. S8:** The results of experiments for the computational time in miliseconds (ms) of critical, redundant and intermittent sets using scale-free networks constructed with *N*=5,000 nodes and average degree <*k*>=3. Note that the computational time in the newly proposed algorithmic method decreases when the degree exponent decreases.


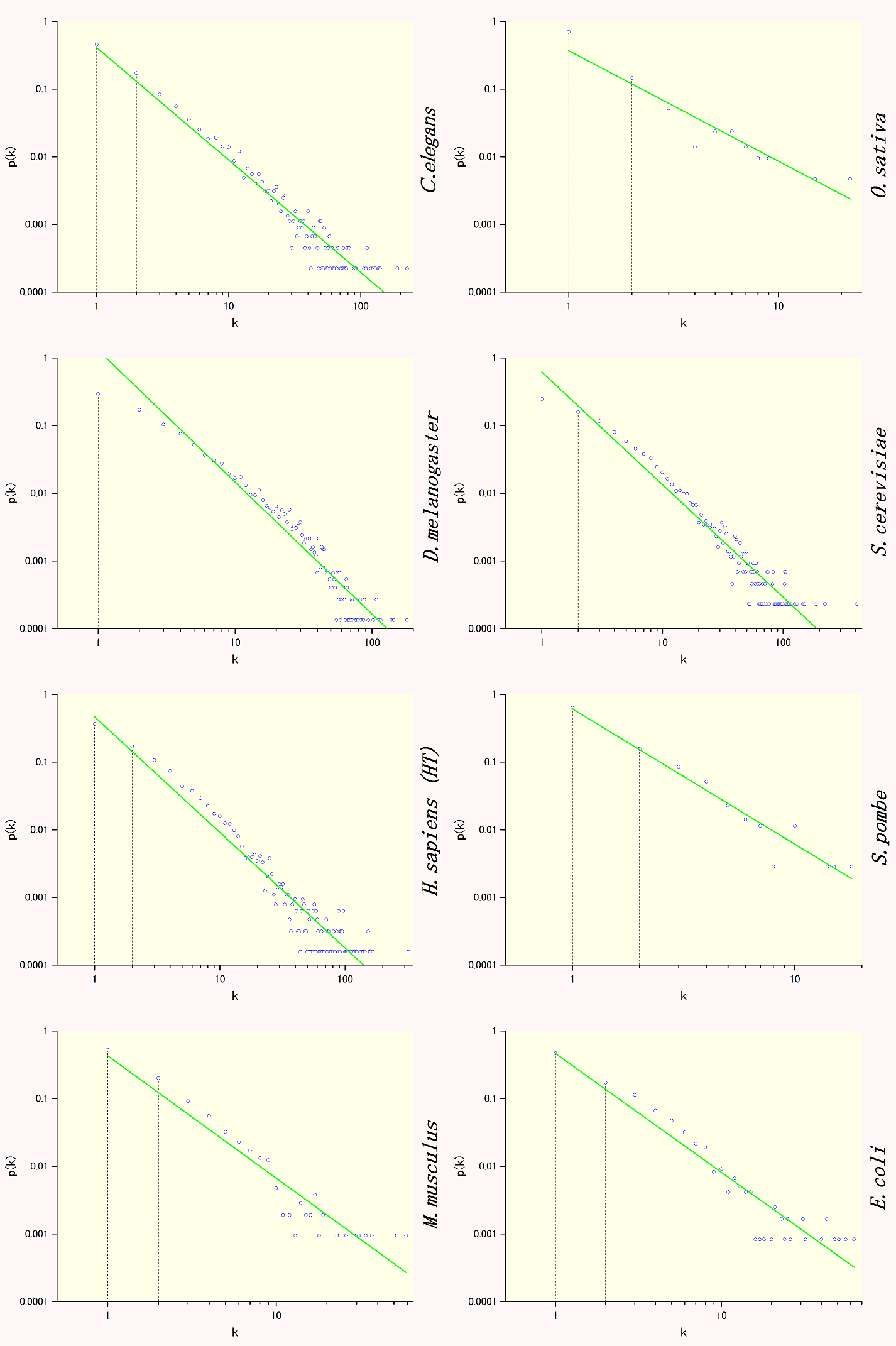


**Fig. S9:** The probability degree distribution of the analysed protein-protein interaction networks. All organisms tend to follow a power-law degree distribution. *S. pombe* and *O. sativa* show the weakest signal because they have the smallest collected datasets for protein number and interactions. Vertical dotted lines highlight the fraction of nodes with degree *k*=1 and *k*=2. Note that scale-free networks show a highly heterogeneous degree distribution, which is very different from that of random networks (see Fig. 1). The newly proposed algorithmic procedure based on proposition 2.2 benefits from the existence of a large number of low-degree nodes and a small number of highly connected nodes.


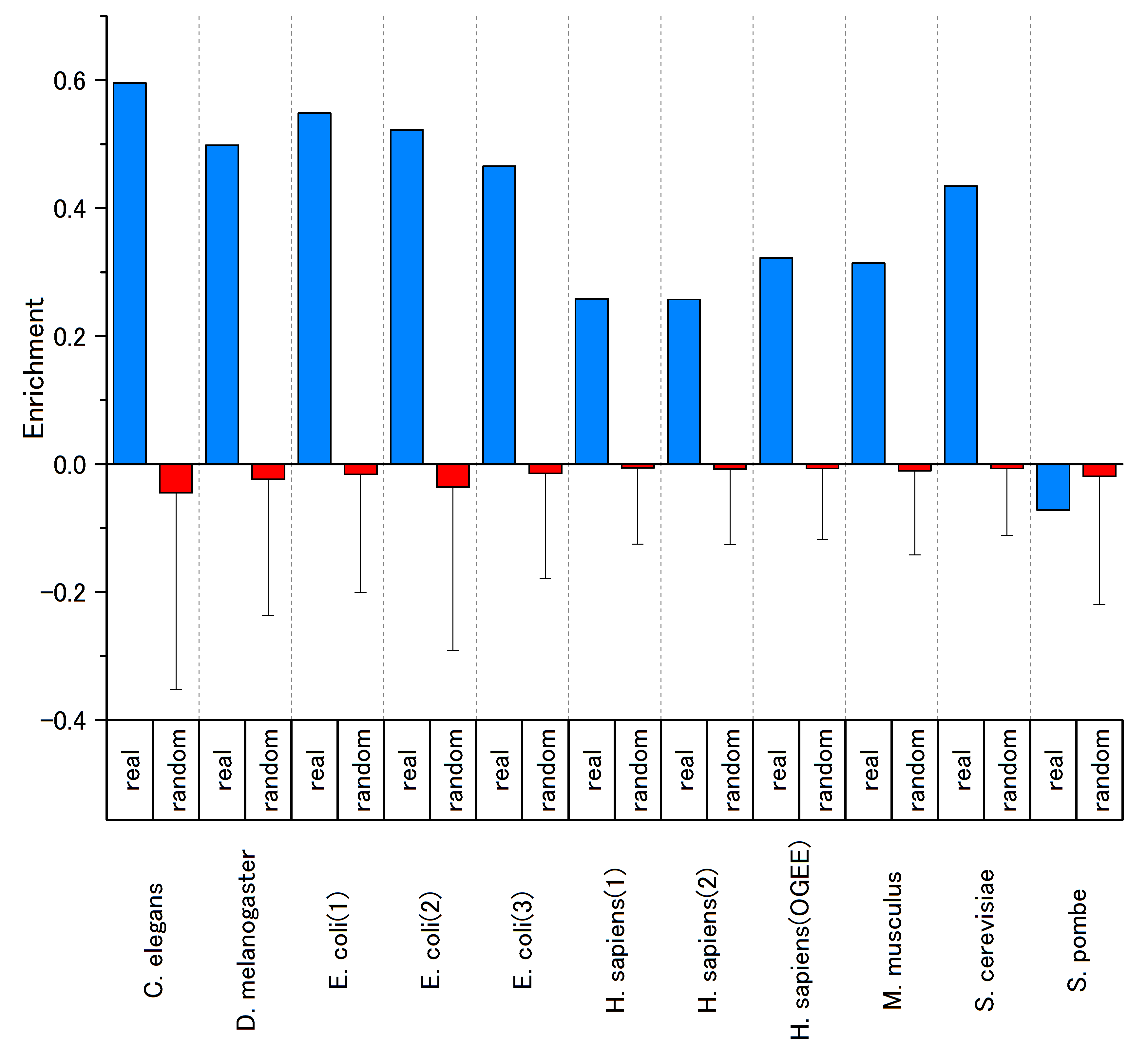


**Fig. S10.** The results show that proteins engaged in critical control are enriched with essential genes for all analysed networks except for that of *S. pombe*, which is not surprising because of the few collected statistics for this organism, as shown in Fig. S9 and Table S1. As a random null model we resampled essential proteins in the network 10,000 times and computed the averaged enrichment and standard error of the mean (s.e.m) as shown in figure.


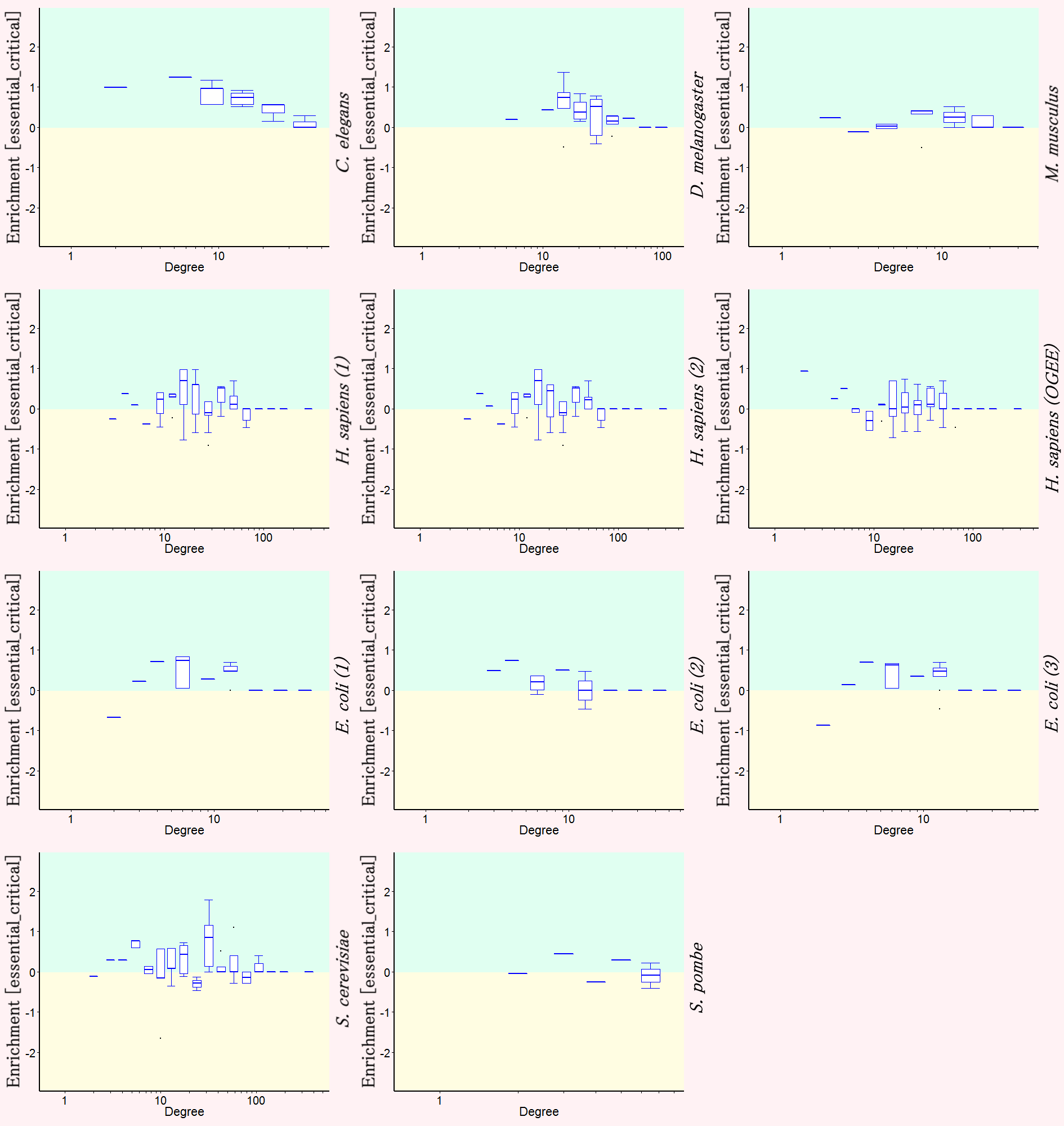


**Fig. S11.** We have defined and computed an enrichment factor that gives information to what extent the critical control accounts for the association with the essentiality, when high degree is controlled. Proteins were classified according to their degree *k* in logarithmic bins of increasing size. For each bin*,* we computed the fraction between the essential proteins with degree *k* and the number of proteins with degree *k* as$f_{k}=N_{k}^{e}/N_{k}$. Next, we calculated the fraction between the essential proteins with degree *k* that are also engaged in critical control (C) $N_{k}^{c,e}$and all proteins with degree *k* engaged in critical control as $f_{k}^{c}=N_{k}^{c,e}/N_{k}^{c}$. Then, the enrichment factor was computed as $E_{k}^{C}=\ln(f_{k}^{C}/f_{k})$. The results are shown using a box-and-whisker plot. See the Methods section for a statistical description of this plot type.


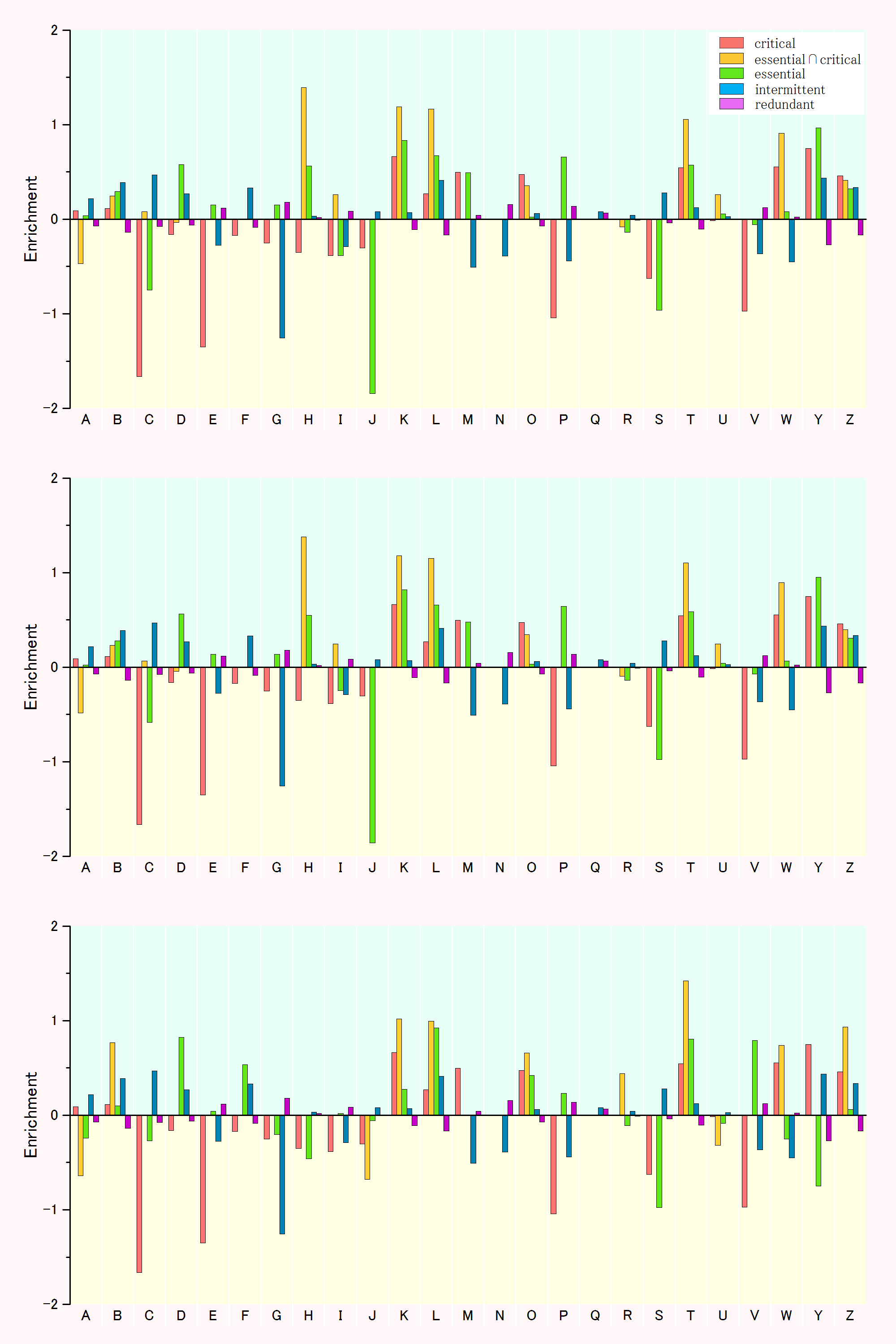


**Fig. S12.** Enrichment factor for each control and essential gene category classified according to the eukaryotic orthologous groups or KOGs. From top to bottom the results correspond to *the H. sapiens (1), H. sapiens (2)* and *H. sapiens (OGEE)* data, respectively. Each letter denotes a functional class as shown in Table 3 and Fig.S13.


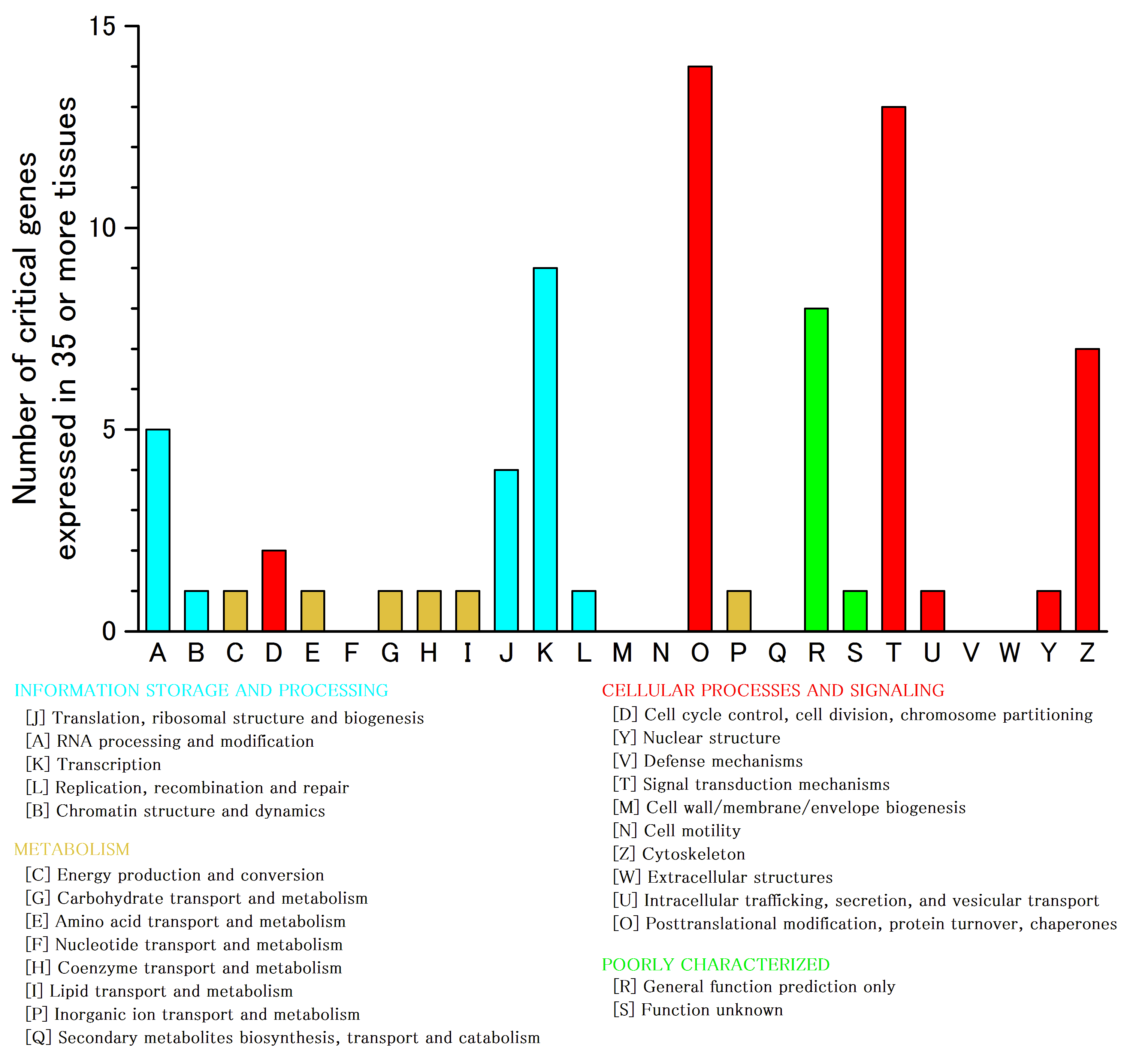


**Fig. S13.** The number of critical genes that are co-expressed in 35 or more healthy human tissues. The genes are classified according to the KOG functional classes. The dataset corresponds to the *H. sapiens (1)* dataset.


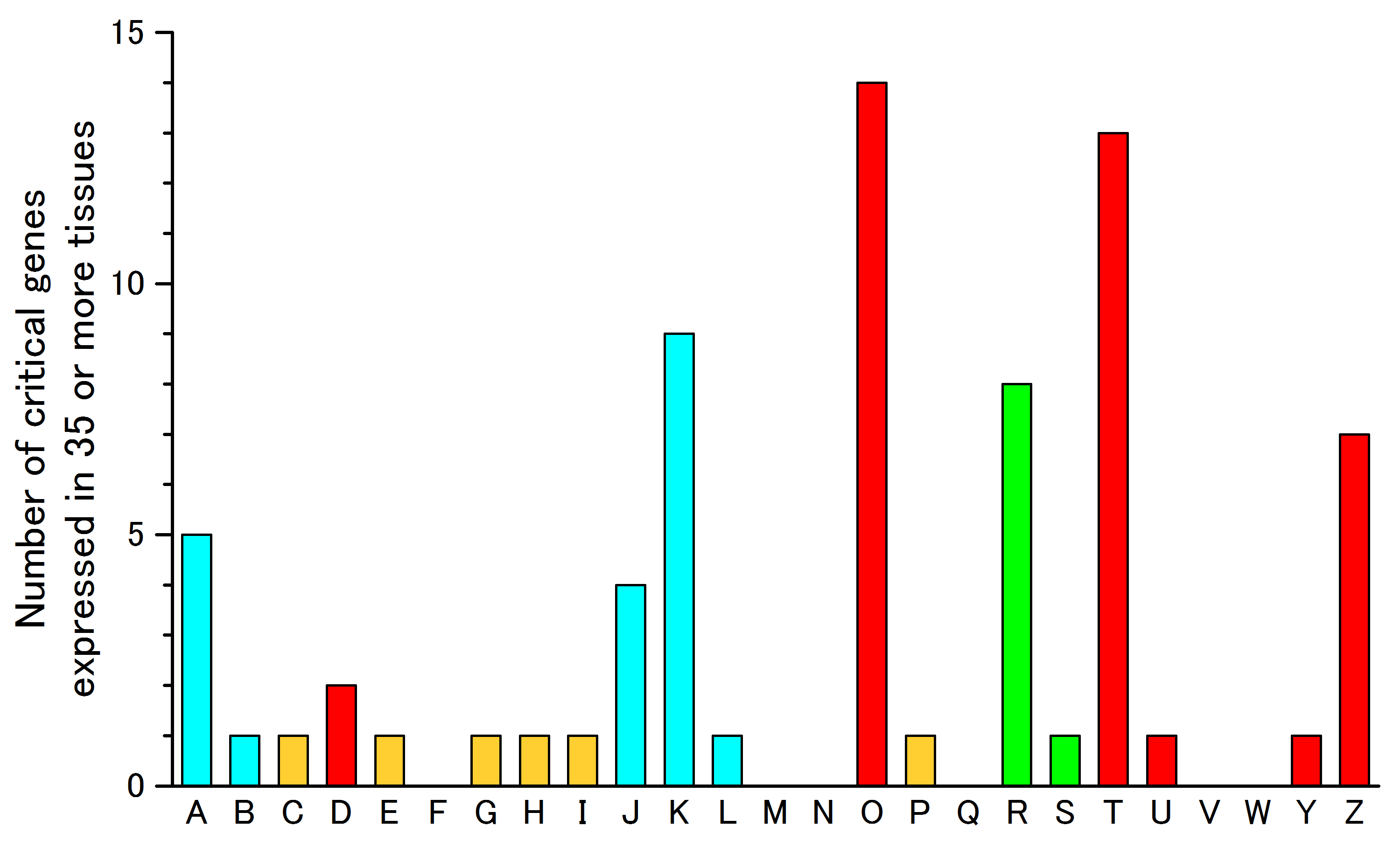


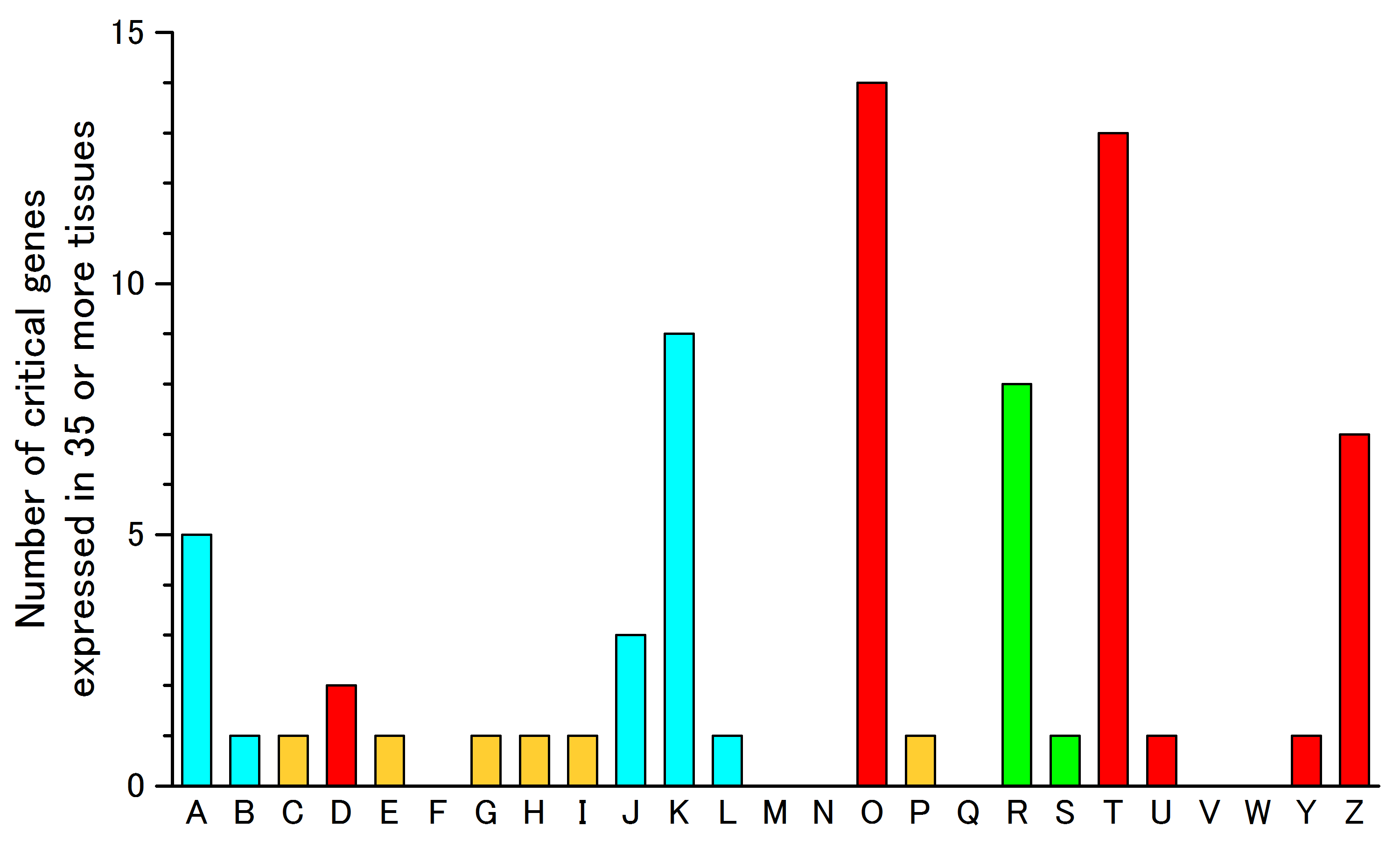


**Fig. S14**: Same as Fig. S13 but for *H. Sapiens (2)* (top) *H. Sapiens (OGEE)* (bottom) datasets. The difference only comes from the functional class J (translational, ribosomal structure and biogenesis).


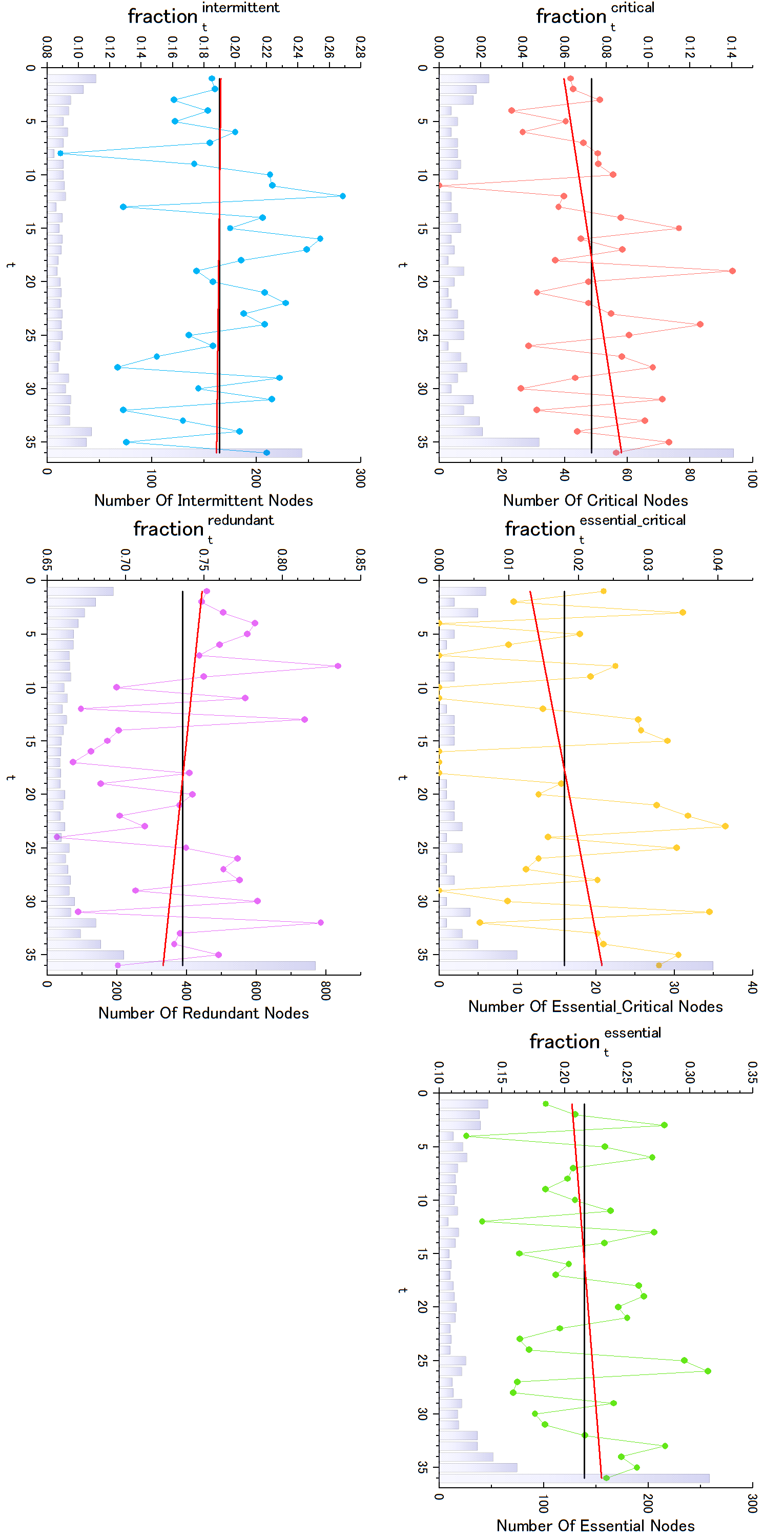


**Fig. S15:** Same as Fig. 6 in main text but for *H. sapiens (2)* dataset for essential genes as shown in Tables 1 and S2.


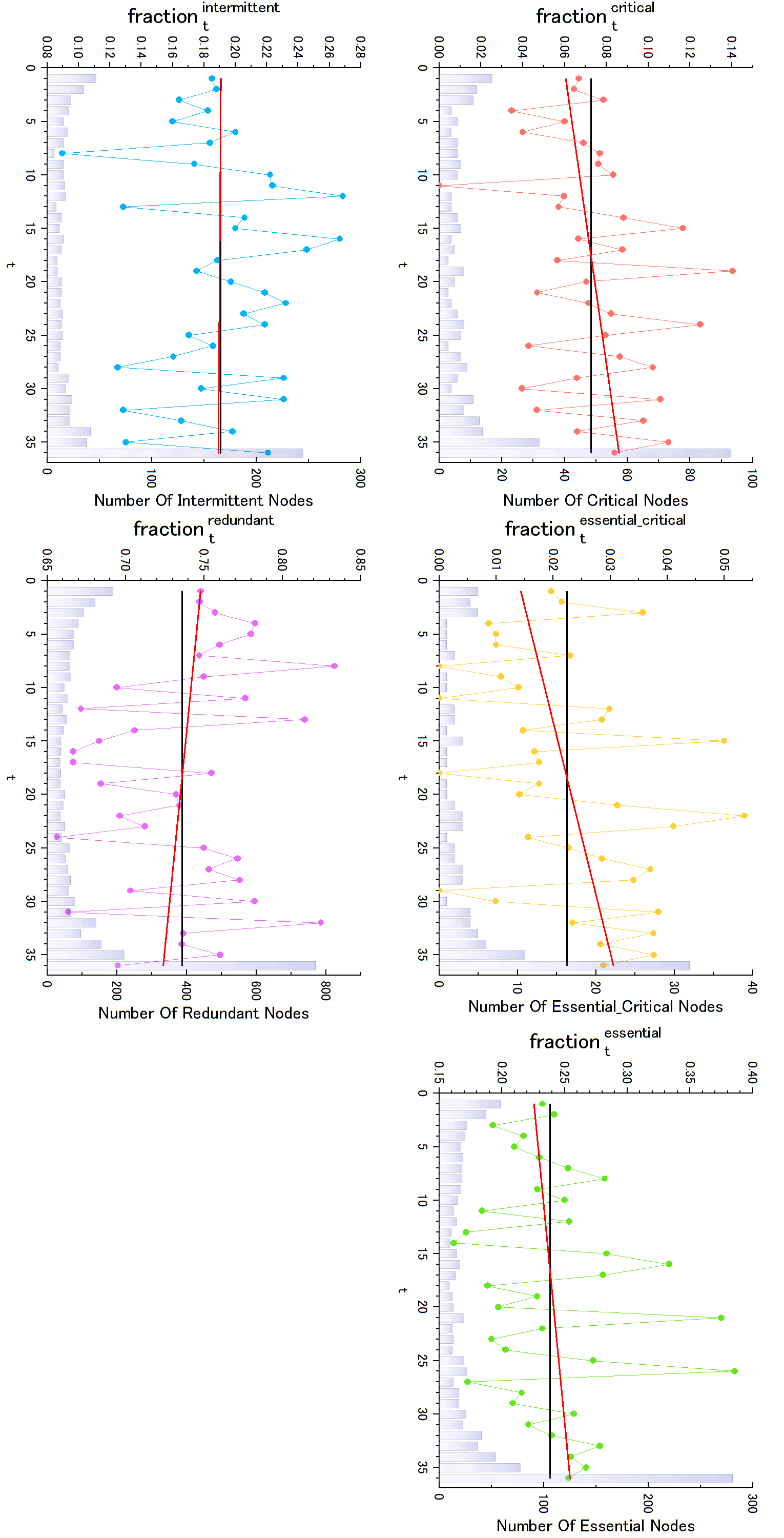


**Fig. S16:** Same as Fig. 6 in main text but for *H. sapiens (OGEE)* dataset for essential genes as shown in Tables 1 and S2.


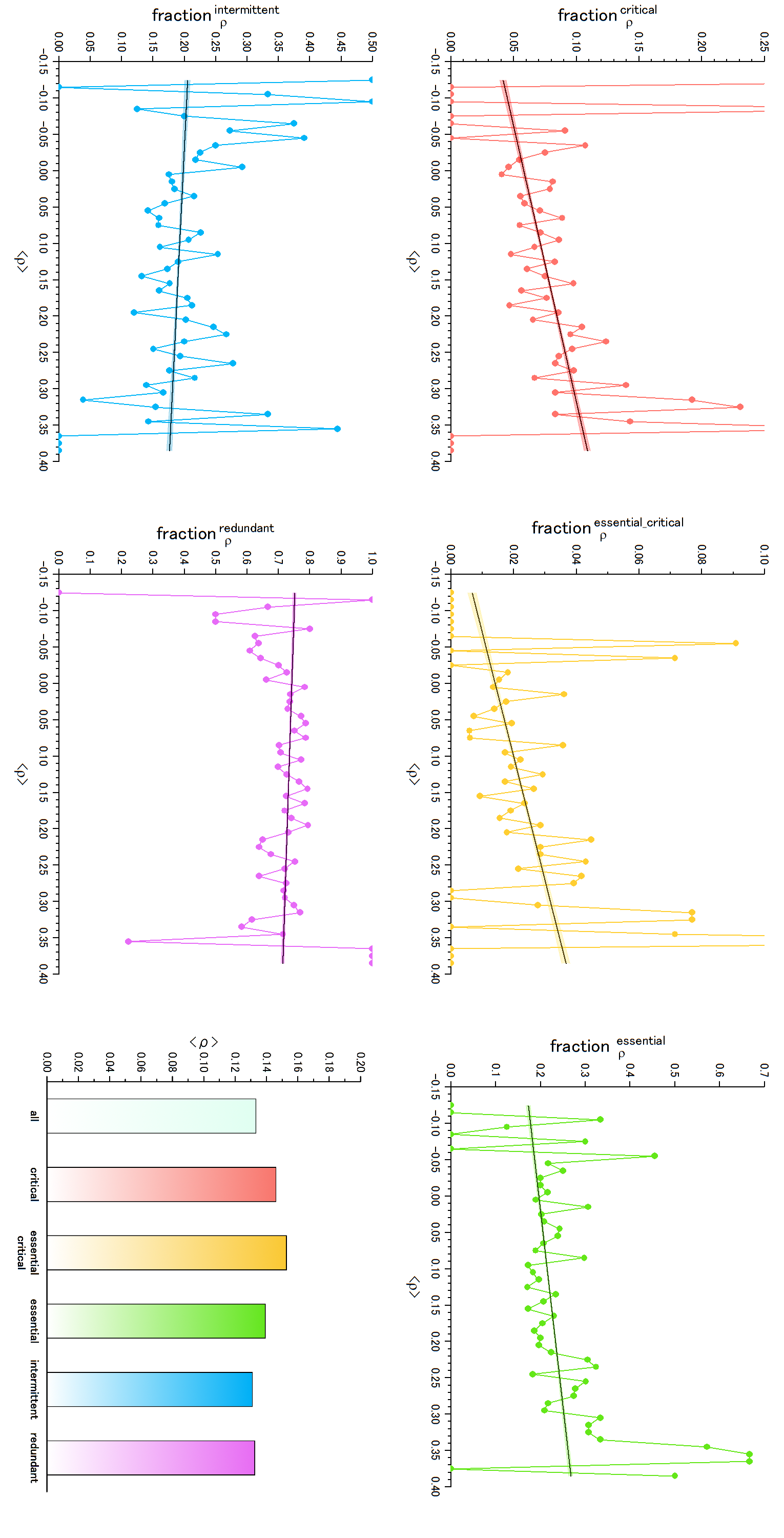


**Fig. S17:** same as Fig. 7 in main text but for *H. sapiens (2)* dataset for essential genes as shown in Tables 1 and S2.


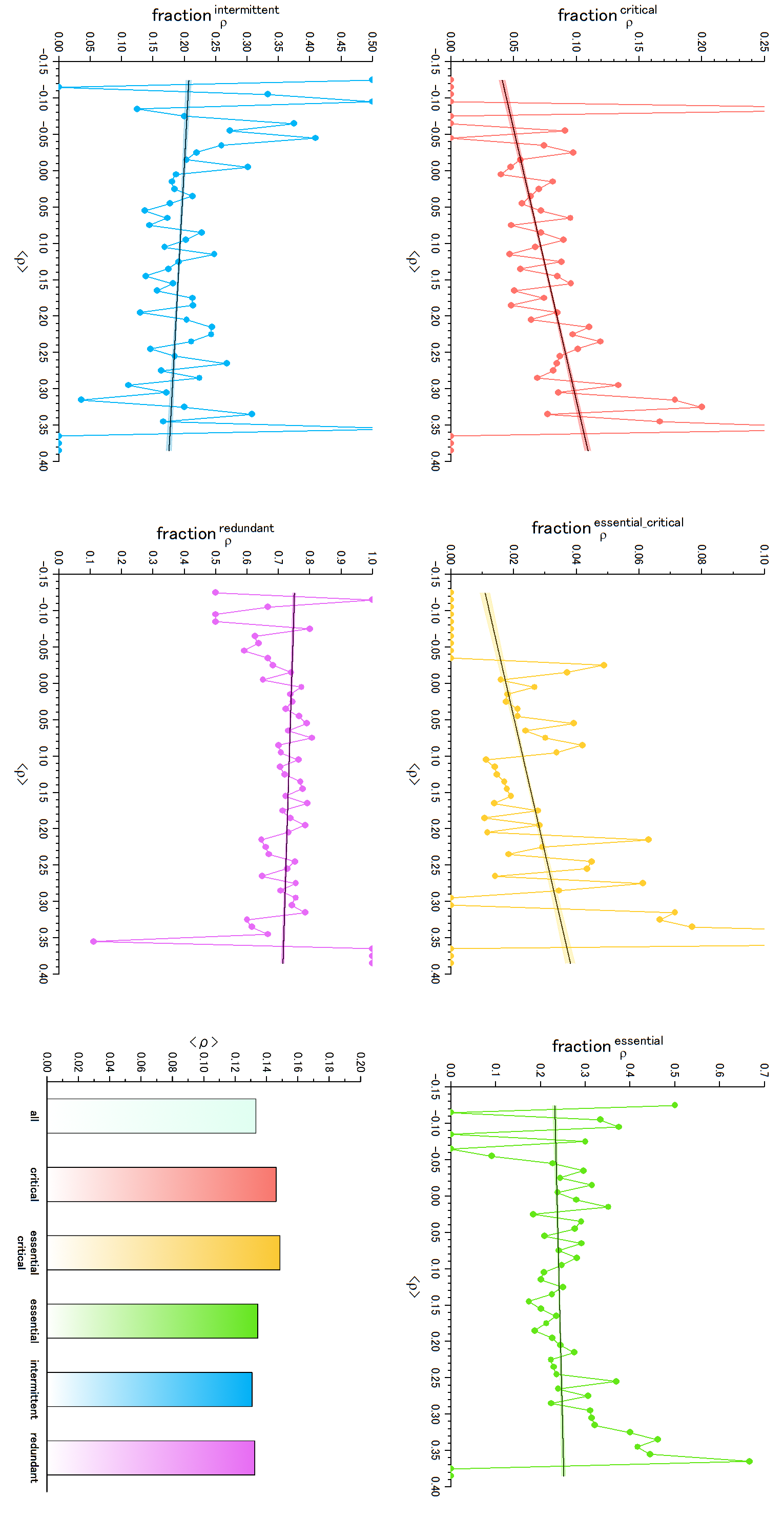


**Fig. S18:** Same as Fig. 7 in main text but for *H. sapiens (OGEE)* dataset for essential genes as shown in Tables 1 and S2.


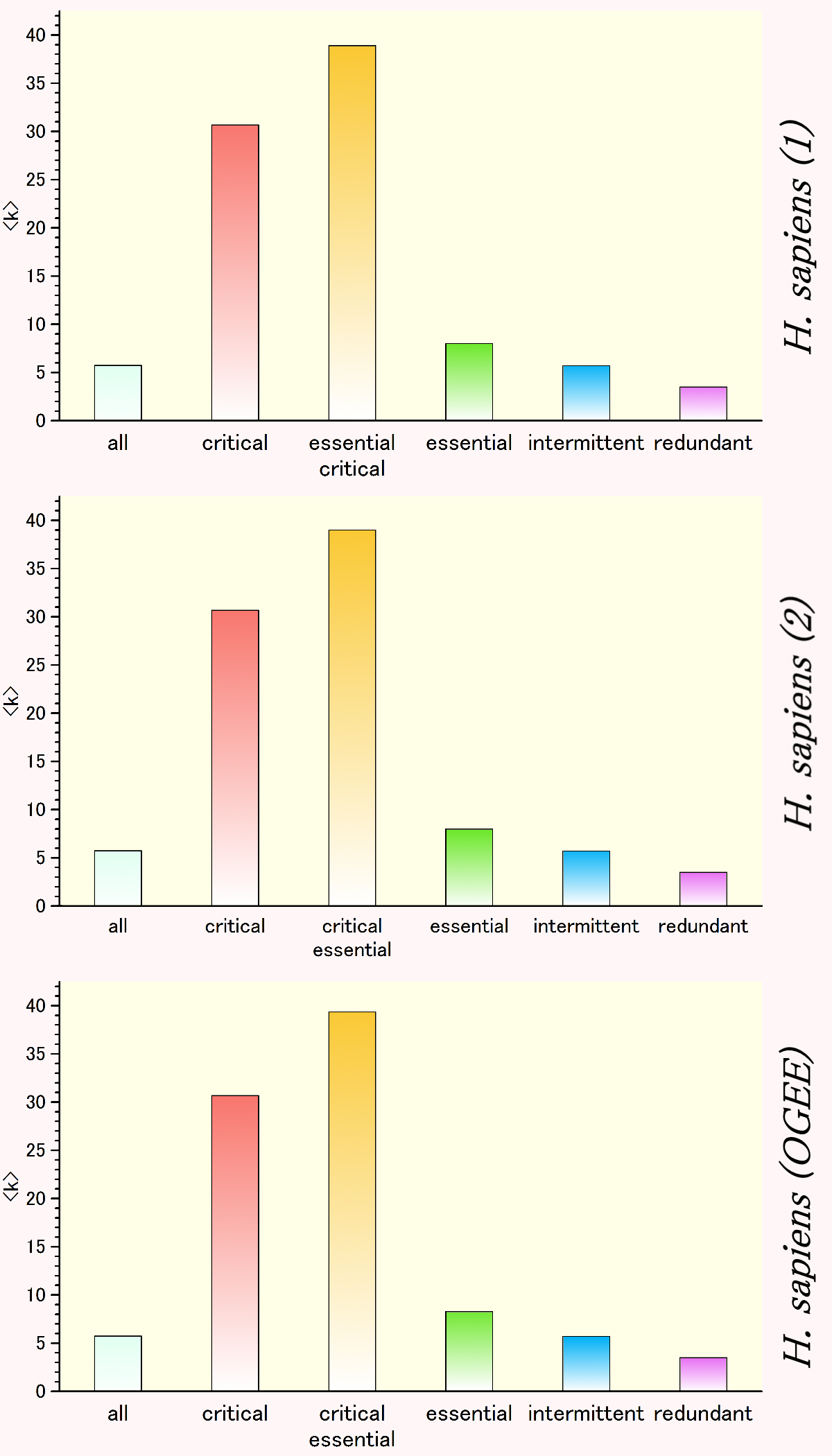


**Fig. S19**: The average degree for proteins in each category. Each figure represents a different essential gene dataset according to Tables 1 and S2. The set asssociated to critical network control and the set that contains essential genes and that are also involved in critical control show the highest average degree.


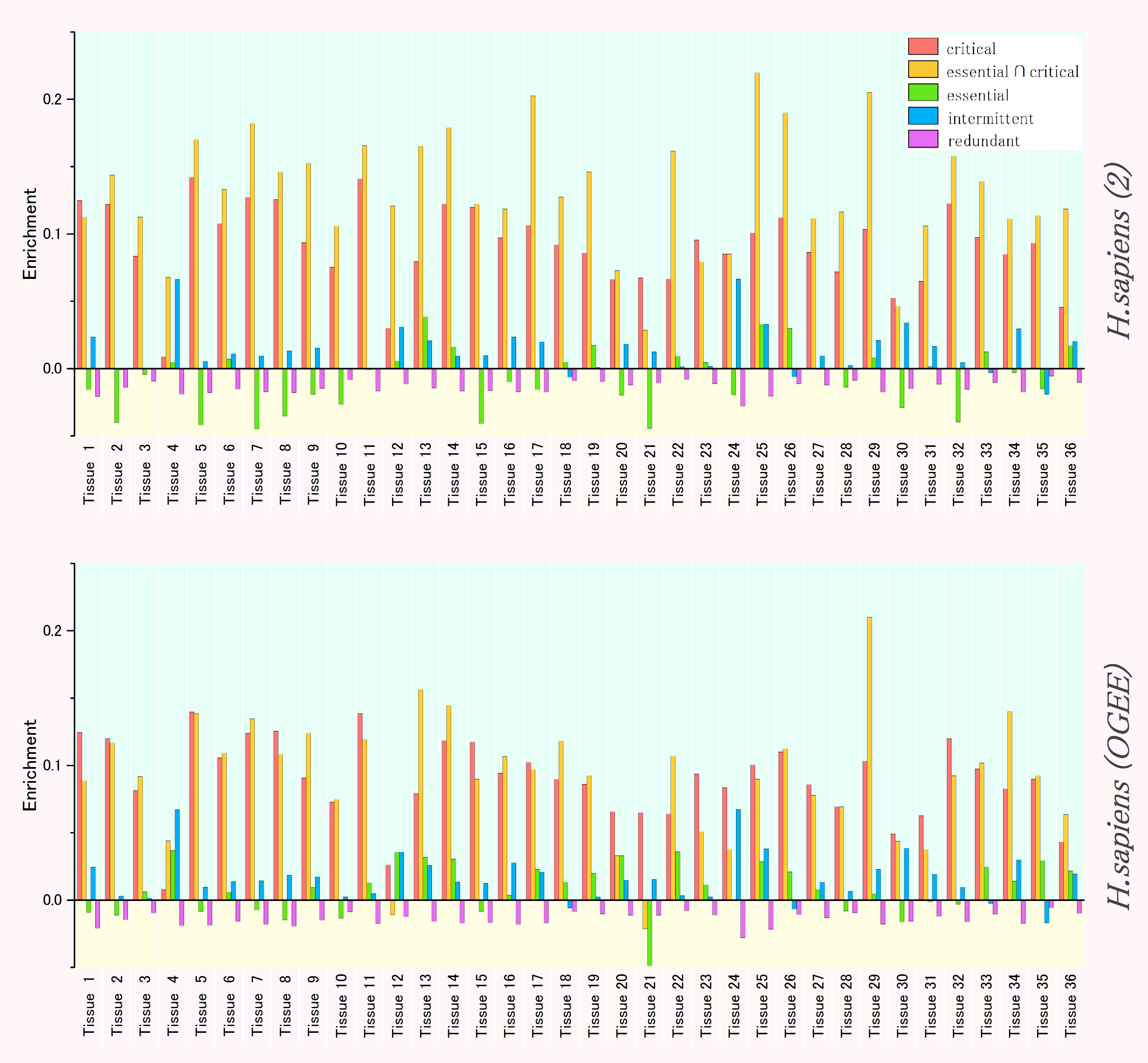


Fig. S20: Same as Fig. 4 but for *H. sapiens* (2) and *H. sapiens (OGEE)* datasets for essential genes.

| **Organism** | **Proteins** | **Interactions** |
| --- | --- | --- |
| *C.elegans* | 4,481 | 11,241 |
| *D.melanogaster* | 7,483 | 25,034 |
| *H.sapiens (HT)* | 6,353 | 18,215 |
| *M.musculus* | 1,055 | 1,400 |
| *O.sativa* | 212 | 201 |
| *S.cerevisiae* | 4,353 | 16,970 |
| *S.pombe* | 351 | 344 |
| *E.coli* | 1,206 | 1,941 |

**Table S1:** The statistics of the analysed protein-protein interaction networks. The data were compiled from the High-quality INTeractomes (HINT) database [20] using binary interactomes for all organisms except *E. coli*. The protein interactions for *H. sapiens* correspond to the high-throughput (HT) interactome. The interactions for *E. coli* were compiled from the results shown in [23].

| **Organism** | **Essential Genes** | **Database** |
| --- | --- | --- |
| *C.elegans* | 294 | DEG |
| *D.melanogaster* | 339 | DEG |
| *H.sapiens 1* | 2452 | DEG |
| *H.sapiens 2* | 2570 | DEG |
| *H.sapiens OGEE* | 4825 | OGEE |
| *M.musculus* | 2114 | DEG |
| *S.cerevisiae* | 1110 | DEG |
| *S.pombe* | 1260 | DEG |
| *E.coli 1* | 609 | DEG |
| *E.coli 2* | 296 | DEG |
| *E.coli 3* | 905 | DEG |

**Table S2:** The number of essential genes collected for each organism and the data source. The essential genes for the analysed organisms were compiled from the Database of Essential Genes (DEG) database [3]. An additional dataset was included in the analysis from OGEE (Online Gene Essentiality Database) (Chen et al., 2011), which is referred to as *H. sapiens (OGEE).* For *E.coli* MG1655, we also compiled several datasets. *E. coli* 1 and 2 refer to data from Gerdes et al. [31] and Baba et al. [30], respectively. Both datasets were available in the DEG. *E. coli* 3 refers to the combined data from *E. coli* 1 and *E. coli* 2.

|  | Network size **15,000** nodes | Network size **25,000** nodes |
| --- | --- | --- |
| *Number of edges* | 30,000 | 50,000 |
| *Computational time (miliseconds)* | 4,762,754 | 20,691,887 |
| *Number of solved ILPs (GLPK)* | 4,186 | 6,859 |

**Table S3:** Results of the new algorithmic computation for critical, redundant and intermittent sets in scale-free networks with $\gamma=2.5$ and average degree *<k>=2* for different network sizes. For these scale-free networks, the new algorithm can exactly solve the critical/redundant NP- hard problem up to 25,000 nodes.

| **Organism** | **Correlation coefficient** | **γ　Degree exponent** | **Error** |
| --- | --- | --- | --- |
| ***C. elegans*** | -0.9557 | 1.6609 | 0.05615 |
| ***D. melanogaster*** | -0.96401 | 1.94945 | 0.05699 |
| ***H. sapiens*** | -0.94468 | 1.70774 | 0.05814 |
| ***M. musculus*** | -0.94252 | 1.80776 | 0.12818 |
| ***O. sativa*** | -0.95329 | 1.63201 | 0.17236 |
| ***S. cerevisiae*** | -0.95395 | 1.66237 | 0.05254 |
| ***S. pombe*** | -0.96783 | 1.99825 | 0.16426 |
| ***E. coli*** | -0.9391 | 1.75517 | 0.11726 |

**Table S4**: The analysed organisms and their degree exponents for each degree distribution

$k^{-\gamma}$.

|  | ***H. sapiens 1 (DEG)*** | ***H. sapiens 2 (DEG)*** | ***H. sapiens OGEE*** |
| --- | --- | --- | --- |
| **Critical** | $(P=1.26\times10^{-155})$ | $(P=2.28\times10^{-150})$ | $(P=1.00\times10^{-167})$ |
| **Essential critical** | $(P=2.62\times10^{-114})$ | $(P=2.28\times10^{-112})$ | $(P=1.15\times10^{-70})$ |
| **Essential** | $(P=1.01\times10^{-99})$ | $(P=1.07\times10^{-89})$ | $(P=1.09\times10^{-5})$ |
| **Intermittent** | $(P=2.37\times10^{-13})$ | $(P=5.71\times10^{-12})$ | $(P=7.94\times10^{-14})$ |
| **Redundant** | $(P=5.41\times10^{-15})$ | $(P=8.05\times10^{-16})$ | $(P=9.49\times10^{-15})$ |

**Table S5:** Two-tailed P-values for the Fisher’s exact test of the correlation observed in Fig. 7 in main text and Figs S17 and S18 in SI.

| GO category | Go term | Number of | | Enrichment | | p-Value | |
| --- | --- | --- | --- | --- | --- | --- | --- |
|  |  | critical | essential  critical | critical | essential  critical | critical | essential  critical |
| Biological  Process | transcription, DNA-templated | 67 | 15 | 0.29 | 0.52 | 1.27E-02 | 4.54E-02 |
| Biological  Process | gene expression | 52 | 13 | 0.42 | 0.77 | 1.59E-03 | 8.04E-03 |
| Biological  Process | positive regulation of transcription from RNA polymerase II promoter | 51 | 21 | 0.59 | 1.44 | 2.71E-05 | 8.82E-09 |
| Biological  Process | innate immune response | 43 | 14 | 0.72 | 1.33 | 3.81E-06 | 1.38E-05 |
| Biological  Process | viral process | 41 | 9 | 0.58 | 0.80 | 2.06E-04 | 1.91E-02 |
| Cellular  Component | cytoplasm | 208 | 45 | 0.35 | 0.55 | 2.62E-10 | 7.90E-06 |
| Cellular  Component | nucleus | 198 | 47 | 0.24 | 0.53 | 1.91E-05 | 6.20E-06 |
| Cellular  Component | cytosol | 155 | 37 | 0.41 | 0.70 | 7.75E-09 | 4.29E-06 |
| Cellular  Component | nucleoplasm | 128 | 36 | 0.26 | 0.73 | 6.77E-04 | 2.83E-06 |
| Cellular  Component | plasma membrane | 87 | 21 | 0.24 | 0.55 | 1.14E-02 | 1.04E-02 |
| Molecular  Function | ATP binding | 63 | 17 | 0.38 | 0.81 | 1.46E-03 | 1.40E-03 |
| Molecular  Function | identical protein binding | 39 | 10 | 0.89 | 1.26 | 1.83E-07 | 4.88E-04 |
| Molecular  Function | sequence-specific DNA binding transcription factor activity | 39 | 13 | 0.36 | 1.00 | 1.78E-02 | 8.25E-04 |
| Molecular  Function | ubiquitin protein ligase binding | 33 | 9 | 1.26 | 1.69 | 8.77E-11 | 3.64E-05 |
| Molecular  Function | ligase activity | 33 | 5 | 0.83 | 0.67 | 5.06E-06 | 1.11E-01 |

**Table S6:** Same as Table 2 for *H. sapiens (2)* dataset

| GO category | Go term | Number of | | Enrichment | | p-Value | |
| --- | --- | --- | --- | --- | --- | --- | --- |
|  |  | critical | essential  critical | critical | essential  critical | critical | essential  critical |
| Biological  Process | transcription, DNA-templated | 67 | 19 | 0.29 | 0.60 | 1.27E-02 | 7.60E-03 |
| Biological  Process | gene expression | 52 | 15 | 0.42 | 0.75 | 1.59E-03 | 4.57E-03 |
| Biological  Process | positive regulation of transcription from RNA polymerase II promoter | 51 | 19 | 0.59 | 1.18 | 2.71E-05 | 3.49E-06 |
| Biological  Process | innate immune response | 43 | 19 | 0.72 | 1.48 | 3.81E-06 | 3.31E-08 |
| Biological  Process | viral process | 41 | 13 | 0.58 | 1.01 | 2.06E-04 | 7.96E-04 |
| Cellular  Component | cytoplasm | 208 | 50 | 0.35 | 0.50 | 2.62E-10 | 2.27E-05 |
| Cellular  Component | nucleus | 198 | 48 | 0.24 | 0.39 | 1.91E-05 | 7.79E-04 |
| Cellular  Component | cytosol | 155 | 41 | 0.41 | 0.65 | 7.75E-09 | 7.44E-06 |
| Cellular  Component | nucleoplasm | 128 | 37 | 0.26 | 0.59 | 6.77E-04 | 1.03E-04 |
| Cellular  Component | plasma membrane | 87 | 30 | 0.24 | 0.75 | 1.14E-02 | 2.68E-05 |
| Molecular  Function | ATP binding | 63 | 20 | 0.38 | 0.81 | 1.46E-03 | 4.62E-04 |
| Molecular  Function | identical protein binding | 39 | 12 | 0.89 | 1.28 | 1.83E-07 | 1.06E-04 |
| ubiquitin protein ligase binding | sequence-specific DNA binding transcription factor activity | 39 | 14 | 0.36 | 0.91 | 1.78E-02 | 1.24E-03 |
| Molecular  Function | ubiquitin protein ligase binding | 33 | 8 | 1.26 | 1.41 | 8.77E-11 | 6.82E-04 |
| Molecular  Function | ligase activity | 33 | 5 | 0.83 | 0.52 | 5.06E-06 | 2.25E-01 |

**Table S7:** Same as Table 2 for *H. sapiens (OGEE)* dataset.

| Function | Function name | Number of critical genes | Gene name |
| --- | --- | --- | --- |
| A | RNA processing and modification | 5 | DDX24, DDX39B, U2AF2, SF3B3, UPF3A |
| B | Chromatin structure and dynamics | 1 | TLE1 |
| C | Energy production and conversion | 1 | ATP6AP1 |
| D | Cell cycle control, cell division, chromosome partitioning | 2 | MOB4, PEA15 |
| E | Amino acid transport and metabolism | 1 | GOT2 |
| F | Nucleotide transport and metabolism | 0 |  |
| G | Carbohydrate transport and metabolism | 1 | GAPDH |
| H | Coenzyme transport and metabolism | 1 | ALAS1 |
| I | Lipid transport and metabolism | 1 | FDFT1 |
| J | Translation, ribosomal structure and biogenesis | 4 | EEF1A1, RPLP1, RPL8, RPS3A |
| K | Transcription | 9 | XBP1, CTBP1, MAX, TCF4, ZNHIT3, TCF12, SKP1, MED23, TCEB1 |
| L | Replication, recombination and repair | 1 | XRCC6 |
| M | Cell wall/membrane/envelope biogenesis | 0 |  |
| N | Cell motility | 0 |  |
| O | Posttranslational modification, protein turnover, chaperones | 14 | UBE2I, UBE2D3, UBE2D2, UBE2D4, RNF11, UBE2E1, UBE2E3, UBE2N, UBE2K, YWHAE, UBE3A, UBE2L6, DNAJA1, CDC34 |
| P | Inorganic ion transport and metabolism | 1 | SAT1 |
| Q | Secondary metabolites biosynthesis, transport and catabolism | 0 |  |
| R | General function prediction only | 8 | EWSR1, CDC42, PLEKHF2, RAC1, LMO4, RAP2A, KLF10, PPFIA1 |
| S | Function unknown | 1 | WBP11 |
| T | Signal transduction mechanisms | 13 | NCK1, FYN, CRK, PIK3R1, ACVR1, MAPK14, NUDT3, ARHGDIA, PSEN1, MPP3, PRKAR1A, MAPK10, PPP2CA |
| U | Intracellular trafficking, secretion, and vesicular transport | 1 | SEC23B |
| V | Defense mechanisms | 0 |  |
| W | Extracellular structures | 0 |  |
| Y | Nuclear structure | 1 | NSFL1C |
| Z | Cytoskeleton | 7 | MAP1LC3B, NDEL1, TUBGCP4, PFN2, GABARAPL2, ARPC3, DYNLL1 |

**Table S8:** Same as Table 3 for *H. sapiens (2)* dataset.

| Function | Function name | Number of critical genes | Gene name |
| --- | --- | --- | --- |
| A | RNA processing and modification | 5 | DDX24, DDX39B, U2AF2, SF3B3, UPF3A |
| B | Chromatin structure and dynamics | 1 | TLE1 |
| C | Energy production and conversion | 1 | ATP6AP1 |
| D | Cell cycle control, cell division, chromosome partitioning | 2 | MOB4, PEA15 |
| E | Amino acid transport and metabolism | 1 | GOT2 |
| F | Nucleotide transport and metabolism | 0 |  |
| G | Carbohydrate transport and metabolism | 1 | GAPDH |
| H | Coenzyme transport and metabolism | 1 | ALAS1 |
| I | Lipid transport and metabolism | 1 | FDFT1 |
| J | Translation, ribosomal structure and biogenesis | 3 | EEF1A1, RPL8, RPS3A |
| K | Transcription | 9 | XBP1, CTBP1, MAX, TCF4, ZNHIT3, TCF12, SKP1, MED23, TCEB1 |
| L | Replication, recombination and repair | 1 | XRCC6 |
| M | Cell wall/membrane/envelope biogenesis | 0 |  |
| N | Cell motility | 0 |  |
| O | Posttranslational modification, protein turnover, chaperones | 14 | UBE2I, UBE2D3, UBE2D2, UBE2D4, RNF11, UBE2E1, UBE2E3, UBE2N, UBE2K, YWHAE, UBE3A, UBE2L6, DNAJA1, CDC34 |
| P | Inorganic ion transport and metabolism | 1 | SAT1 |
| Q | Secondary metabolites biosynthesis, transport and catabolism | 0 |  |
| R | General function prediction only | 8 | EWSR1, CDC42, PLEKHF2, RAC1, LMO4, RAP2A, KLF10, PPFIA1 |
| S | Function unknown | 1 | WBP11 |
| T | Signal transduction mechanisms | 13 | NCK1, FYN, CRK, PIK3R1, ACVR1, MAPK14, NUDT3, ARHGDIA, PSEN1, MPP3, PRKAR1A, MAPK10, PPP2CA |
| U | Intracellular trafficking, secretion, and vesicular transport | 1 | SEC23B |
| V | Defense mechanisms | 0 |  |
| W | Extracellular structures | 0 |  |
| Y | Nuclear structure | 1 | NSFL1C |
| Z | Cytoskeleton | 7 | MAP1LC3B, NDEL1, TUBGCP4, PFN2, GABARAPL2, ARPC3, DYNLL1 |

**Table S9:** Same as Table 3 for *H. sapiens (OGGE)* dataset.
